# TRIM5α requires Ube2W to anchor Lys63-linked ubiquitin chains and restrict reverse transcription

**DOI:** 10.15252/embj.201490361

**Published:** 2015-06-22

**Authors:** Adam J Fletcher, Devin E Christensen, Chad Nelson, Choon Ping Tan, Torsten Schaller, Paul J Lehner, Wesley I Sundquist, Greg J Towers

**Affiliations:** 1 MRC Centre of Medical Molecular Virology, Division of Infection and Immunity, University College LondonLondon, UK; 2 Department of Biochemistry and HSC Core Facilities, University of Utah School of MedicineSalt Lake City, UT, USA; 3 Cambridge Institute for Medical Research, Department of Medicine, University of CambridgeCambridge, UK

**Keywords:** restriction, TRIM5α, Ube2N, Ube2W, ubiquitin

## Abstract

TRIM5α is an antiviral, cytoplasmic, E3 ubiquitin (Ub) ligase that assembles on incoming retroviral capsids and induces their premature dissociation. It inhibits reverse transcription of the viral genome and can also synthesize unanchored polyubiquitin (polyUb) chains to stimulate innate immune responses. Here, we show that TRIM5α employs the E2 Ub-conjugating enzyme Ube2W to anchor the Lys63-linked polyUb chains in a process of TRIM5α auto-ubiquitination. Chain anchoring is initiated, in cells and *in vitro*, through Ube2W-catalyzed monoubiquitination of TRIM5α. This modification serves as a substrate for the elongation of anchored Lys63-linked polyUb chains, catalyzed by the heterodimeric E2 enzyme Ube2N/Ube2V2. Ube2W targets multiple TRIM5α internal lysines with Ub especially lysines 45 and 50, rather than modifying the N-terminal amino group, which is instead αN-acetylated in cells. E2 depletion or Ub mutation inhibits TRIM5α ubiquitination in cells and restores restricted viral reverse transcription, but not infection. Our data indicate that the stepwise formation of anchored Lys63-linked polyUb is a critical early step in the TRIM5α restriction mechanism and identify the E2 Ub-conjugating cofactors involved.

## Introduction

TRIM5α is an innate immune effector that belongs to the tripartite (TRIM) protein superfamily. The conserved tripartite architecture comprises a really interesting new gene (RING) E3 ubiquitin (Ub) ligase domain, one or two B-box domains, and a coiled-coil, followed by variable protein interaction domain(s). Although TRIM proteins can have wide-ranging biological roles, anti-pathogen defense functions are common (Nisole *et al*, 2005; McNab *et al*, 2011). The expression of many TRIM proteins is induced by type I interferons, and several have been shown to manipulate immune signaling pathways by ubiquitinating signal transducing proteins, such as TRIM23, TRIM25, and TRIM56 (Gack *et al*, 2007; Arimoto *et al*, 2010; Tsuchida *et al*, 2010). Certain TRIM proteins, including TRIM5α (Stremlau *et al*, 2004), TRIM21 (Mallery *et al*, 2010), and TRIM56 (Wang *et al*, 2011), have also been shown to target viral replication specifically. An emerging theme is that antiviral TRIM proteins both inhibit viral infection and initiate innate immune signaling cascades that lead to inflammatory cytokine production and an antiviral state (Pertel *et al*, 2011; McEwan *et al*, 2013; Uchil *et al*, 2013; Versteeg *et al*, 2013). The molecular mechanisms that underlie these multiple activities are not yet well defined and are of great interest for understanding the innate immune detection of viruses.

TRIM5α represents an important barrier to zoonotic retroviral infection (Stremlau *et al*, 2004; Wu *et al*, 2013, 2015). Multimers of homodimeric TRIM5α bind susceptible retroviral capsids in the cytoplasm, inducing premature capsid dissociation and inhibiting reverse transcription (Stremlau *et al*, 2006; Diaz-Griffero *et al*, 2009; Black & Aiken, 2010; Ganser-Pornillos *et al*, 2011; Zhao *et al*, 2011; Goldstone *et al*, 2014). Several observations indicate the involvement of the Ub-proteasome system in these processes: (i) proteasome inhibitors restore slow capsid dissociation kinetics and rescue formation of integration-competent reverse transcripts, but do not restore viral infectivity (Anderson *et al*, 2006; Wu *et al*, 2006; Kutluay *et al*, 2013); (ii) TRIM5α and proteasomal components co-localize in cells (Campbell *et al*, 2008; Lukic *et al*, 2011; Danielson *et al*, 2012); and (iii) the short half-life of TRIM5α is decreased even further upon recognition of restriction-sensitive capsids (Rold & Aiken, 2008), and lengthened by proteasome inhibition (Diaz-Griffero *et al*, 2006). Thus, one plausible model for restriction is that TRIM5α–capsid complexes are diverted into a constitutive, proteasome-dependent unfolding/degradation pathway (Towers, 2007), although an alternative autophagosomal unfolding/degradation model has also been suggested (Mandell *et al*, 2014). In addition to proteasome recruitment, TRIM5α has been shown to generate unanchored Lys63-linked polyubiquitin (polyUb) chains upon binding to susceptible retroviral capsids. These unanchored polyUb chains are postulated to activate transforming growth factor-β-activated kinase 1 (TAK1), which in turn stimulates NF-κB nuclear translocation and induces antiviral gene expression (Shi *et al*, 2008; Pertel *et al*, 2011).

The Ub enzymology underlying different TRIM5α activities remains to be defined. Toward this end, we have employed genetic and biochemical approaches to identify and characterize two E2 Ub-conjugating enzymes required for TRIM5α restriction of retroviral reverse transcription: Ube2W and the heterodimeric Ube2N/Ube2V2. We find that Ube2W can attach single Ub molecules directly to TRIM5α, which can then act as substrates for Ube2N/Ube2V2 in the synthesis of TRIM5α-anchored Lys63-linked polyUb chains, both *in vitro* and in cells. We find that TRIM5α is αN-acetylated in cells, a modification that blocks N-terminal ubiquitination, and that Ube2W targets specific TRIM5α lysine residues with Ub *in vitro*. Furthermore, Ube2W, Ube2N/Ube2V2, and the Ub residue Lys63 are each required for TRIM5α restriction of viral DNA synthesis, but not infection. Thus, our data indicate that TRIM5α employs multiple E2 enzymes to synthesize protein-anchored Ub chains that are required for inhibition of retroviral reverse transcription.

## Results

### TRIM5α requires two non-redundant E2 enzymatic activities to inhibit retroviral DNA synthesis

**Figure 1:**
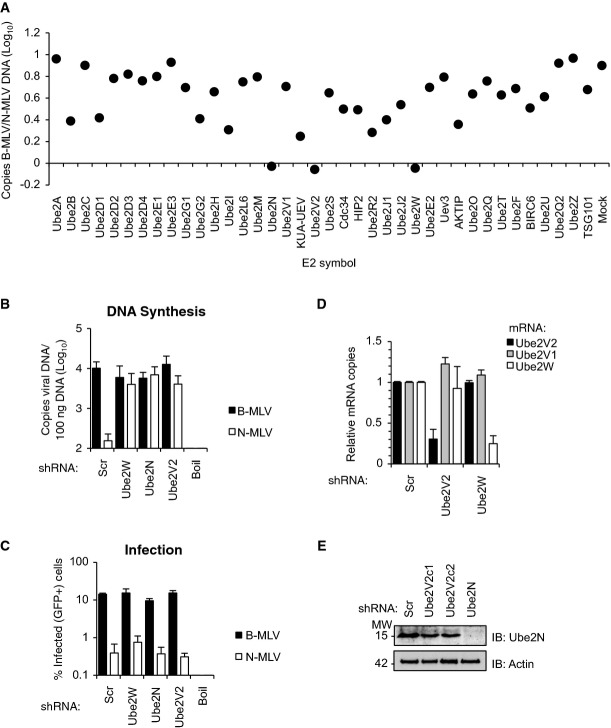
TRIM5α restriction of N-MLV reverse transcription requires the E2 enzymes Ube2W, Ube2N, and Ube2V2 A siRNA screen against 38 human E2 Ub-conjugating enzymes in HeLa cells identifies three enzymes necessary for the block to N-MLV reverse transcription. HeLa cells in duplicate 96-well plates were reverse-transfected with siRNA SMARTpools at a final concentration of 30 nM. Cells were washed 24 h post-transfection and then incubated for additional 24 h. Cells were then infected with either N-tropic or B-tropic MLV-GFP vector expressing GFP at MOI ˜0.2. At 6 h post infection (p.i.), cells were lysed, total DNA was purified, and copies of viral DNA (TaqMan GFP qPCR) were determined and plotted as the ratio of B-MLV:N-MLV copies/100 ng total DNA. Values are means of triplicate experiments.

We performed an RNAi screen to identify non-redundant E2 Ub-conjugating enzymes required for TRIM5α antiviral activity. Because proteasome inhibitors restore retroviral reverse transcription without rescuing viral infectivity (Anderson *et al*, 2006; Wu *et al*, 2006; Kutluay *et al*, 2013), we reasoned that abrogating functionally important ubiquitination activities associated with TRIM5α should rescue reverse transcription, but might not rescue viral infectivity. Human TRIM5α (TRIM5α_hu_) acts differently on two closely related variants of murine leukemia virus (MLV) that differ in their capsid sequences: TRIM5α_hu_ restricts N-tropic MLV (N-MLV) but not B-tropic MLV (B-MLV) (Towers *et al*, 2000; Besnier *et al*, 2003; Keckesova *et al*, 2004; Perron *et al*, 2004; Yap *et al*, 2004). B-MLV therefore serves as a convenient control for differentiating between loss of TRIM5α_hu_-dependent E2 activities, which should specifically affect N-MLV infection, versus alterations in cell viability or metabolism, which should affect both N-MLV and B-MLV. We used single-round, VSV-G-pseudotyped N- and B-MLV vectors expressing GFP to assess endogenous TRIM5α_hu_ activity. As expected, these two viral vectors were equally infectious in feline CrFK cells, which do not express a restricting TRIM5α protein (McEwan *et al*, 2009) (Supplementary Fig S1A), but were differentially infectious in human HeLa cells, which endogenously express TRIM5α (Fig1A, Mock). siRNAs targeting 38 different human E2 or E2 variant (UEV) proteins, which collectively encompass the majority of known human E2 enzymes (Markson *et al*, 2009), were then tested for their ability to restore N-MLV DNA synthesis (Fig1A). The three strongest hits were Ube2W, Ube2N (Ubc13), and Ube2V2 (Mms2); individual depletions of any of these E2 proteins abrogated TRIM5α_hu_-dependent restriction of N-MLV DNA synthesis completely. These three proteins likely represent just two different E2 enzymatic activities because Ube2N and Ube2V2 function together in a heterodimeric E2 enzyme complex comprising an active Ube2N E2 subunit and a catalytically inactive Ube2V2 subunit that provides specificity for synthesis of Lys63-linked polyUb chains (Hofmann & Pickart, 1999; McKenna *et al*, 2003).

B, C TE671 cells that stably expressed Ube2W-, Ube2V2-, or Ube2N-specific shRNA, or a scrambled shRNA (Scr) were transduced with N- or B-MLV-GFP vectors. Copies of viral DNA at 6 h post infection (p.i.) (TaqMan GFP qPCR) (B) and percent infection at 48 h p.i. (flow cytometry for GFP) (C) were determined in parallel samples. Boiled virus served as a negative control for plasmid contamination. Mean ± SEM, *n* = 3. See also Supplementary Figure S1.

D mRNA levels of Ube2V2, Ube2V1, or Ube2W were assessed by RT–qPCR in TE671 cells that stably expressed Ube2V2- or Ube2W-specific shRNA or Scr. Values were calculated relative to GAPDH mRNA and expressed relative to levels in cells expressing Scr control. Mean ± SEM of triplicates.

E Immunoblot (IB) detecting Ube2N in two cell lines expressing Ube2V2-specific shRNA (V2c1 and V2c2) or cells expressing Ube2N-specific shRNA.

The strongest hits from the siRNA screen were validated by individually depleting Ube2W, Ube2N, and Ube2V2 from restrictive human TE671 cells using shRNA stably expressed from retroviral vectors (Fletcher *et al*, 2010). E2-depleted cells were infected with equivalent doses of N- or B-MLV, and viral DNA synthesis and infectivity were measured 6 and 48 h postinfection, respectively. Cells expressing a scrambled control shRNA restricted N-MLV efficiently, as measured by strong reductions in DNA synthesis (Fig1B) and infectivity (Fig1C). However, depleting either Ube2W, Ube2N, or Ube2V2 substantially rescued N-MLV DNA synthesis, without major effects on unrestricted B-MLV DNA synthesis (Fig1B). As predicted, E2 depletion rescued DNA synthesis more effectively than it rescued viral infectivity, validating the logic of the screen readout [compare Fig1C (infection) with B (DNA synthesis)]. Specific depletion was confirmed by RT–qPCR for Ube2W and Ube2V2 (Fig1D and Supplementary Fig S1B) or immunoblot (IB) for Ube2N (Fig1E). Because Ube2N can function with either Ube2V2 or Ube2V1 (Uev1a) (Hofmann & Pickart, 1999), we confirmed that depletion of Ube2V1 did not rescue restricted N-MLV DNA synthesis in TE671 cells (Supplementary Fig S1C and D). Furthermore, depletion of Ube2V2 had no impact on the expression of either Ube2V1 (Fig1D) or Ube2N (Fig1E). These experiments indicate that both Ube2W and Ube2N/Ube2V2 are required for efficient restriction of retroviral reverse transcription by TRIM5α_hu_.

### TRIM5α can be ubiquitinated and degraded in a proteasome-dependent fashion

**Figure 2:**
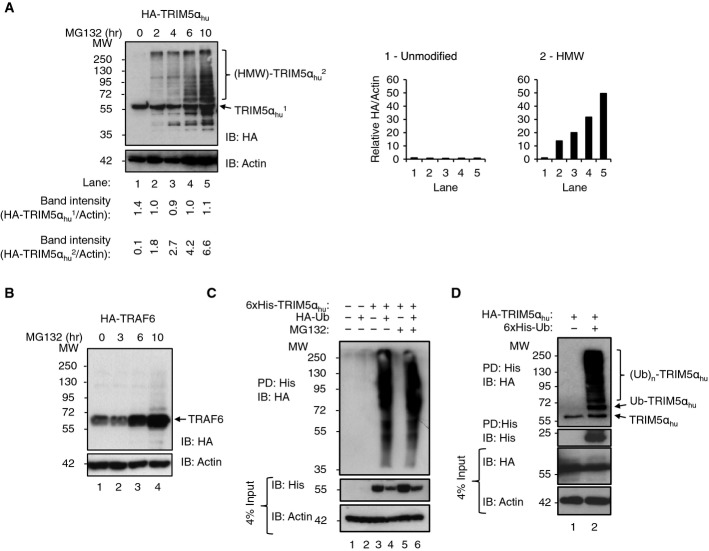
Constitutively ubiquitinated TRIM5α is a proteasome substrate A, B Proteasome inhibition reveals TRIM5α_hu_ high molecular weight (HMW) species. TE671 cells that expressed HA-TRIM5α_hu_ (A) or a control RING protein, HA-TRAF6 (B), were treated with the proteasome inhibitor MG132, lysed at the indicated time intervals, and analyzed by IB detection of the HA-tag (HA), or β-actin (loading control). The band intensity ratios (ImageJ) (HA:β-actin) of unmodified HA-TRIM5α_hu_ (labeled “1”) and HMW HA-TRIM5α_hu_ (labeled “2”) are displayed beneath and normalized to the 0-h time point and plotted.

We next analyzed the ubiquitination state of TRIM5α_hu_ under conditions of proteasome inhibition. We treated TE671 cells exogenously expressing HA-TRIM5α_hu_ with the proteasome inhibitor MG132, and HA-TRIM5α_hu_ species were detected by IB against the HA-tag (Fig2A). A 2-hour MG132 treatment leads to accumulation of higher molecular weight (HMW) HA-TRIM5α_hu_ species (Fig2A, lane 2). The HMW products continued to increase with time, ultimately becoming the predominant TRIM5α species after 10 h of MG132 treatment (Fig2A, lane 5). Measurement of the HA band densities corresponding to the unmodified and HMW forms of HA-TRIM5α_hu_ demonstrated that it is not the unmodified form that accumulated in these experiments (Fig2A); rather it is the HMW species that accumulate, consistent with post-translational modification, such as ubiquitination. We assume their accumulation reflects a lack of degradation owing to proteasome inhibition.

C, D Constitutive TRIM5α_hu_ autoubiquitination can also be detected in the absence of MG132. 293T cells were transfected with the designated combinations of expression constructs for 6×His-TRIM5α_hu_ and HA-Ub. After 48 h, 6×His-TRIM5α_hu_ was pulled down (PD) with a Ni^2+^ affinity matrix, followed by analysis of the input and affinity-purified samples by IB with the designated antibodies (C). TE671 cells that stably expressed HA-TRIM5α_hu_ were mock-transfected or transfected with a construct expressing 6×His-Ub. Cells were not treated with MG132. After 48 h, 6×His-Ub was affinity-purified and the samples were analyzed by IB (D). Antibodies were against the HA-tag (HA), the His-tag (His), or β-actin (loading control). Note some unmodified TRIM5α_hu_ precipitates with the Ni^2+^ affinity matrix in a 6×His-Ub-independent manner. Data information: All blots are representative of at least three independent experiments.

To control for non-specific effects of overexpressing a RING domain-containing protein in cells, we also performed parallel control experiments on TE671 expressing HA-TRAF6, another RING E3 ligase involved in innate immune signaling (Xia *et al*, 2009). In contrast to TRIM5α_hu_, only very small amounts of HMW HA-TRAF6 species accumulated upon MG132 treatment, whereas the unmodified form accumulated (Fig2B), suggesting that HMW accumulation is not a general property of overexpressing an active RING E3 ligase. Our observations are consistent with a model in which TRIM5α_hu_ is post-translationally modified, leading to accumulation of HMW species that are normally turned over in a proteasome-dependent fashion. This result should be treated with some caution, however, because proteasome inhibition, and consequent Ub depletion, can also affect other Ub-dependent cellular processes.

To confirm that the HMW TRIM5α_hu_ species were ubiquitination products, we co-transfected 293T cells with 6×His-tagged TRIM5α_hu_ (His-TRIM5α_hu_) and HA-tagged Ub (HA-Ub). His-TRIM5α_hu_ species were enriched by Ni^2+^ affinity purification, and the HMW His-TRIM5α_hu_ species were confirmed as ubiquitinated products by IB, detecting the HA-tag (Fig2C, lanes 4 and 6). Importantly, in this case, ubiquitinated His-TRIM5α_hu_ species were detectable in the absence of MG132 treatment (lane 4). We assume that these species were enriched by the affinity purification step and could therefore be detected by IB. Ubiquitinated TRIM5α_hu_ ((Ub)_n_-TRIM5α) was also detected in a reciprocal affinity purification (pulldown of His-Ub, IB detection of HA-TRIM5α_hu_), again in the absence of MG132 treatment (Fig2D). These experiments demonstrate that TRIM5α can be heavily ubiquitinated in cells, in good agreement with some previous studies (Diaz-Griffero *et al*, 2006), but not others (Rold & Aiken, 2008).

### Depletion of Ube2W or Ube2N/Ube2V2 suppresses TRIM5α ubiquitination in cells

**Figure 3:**
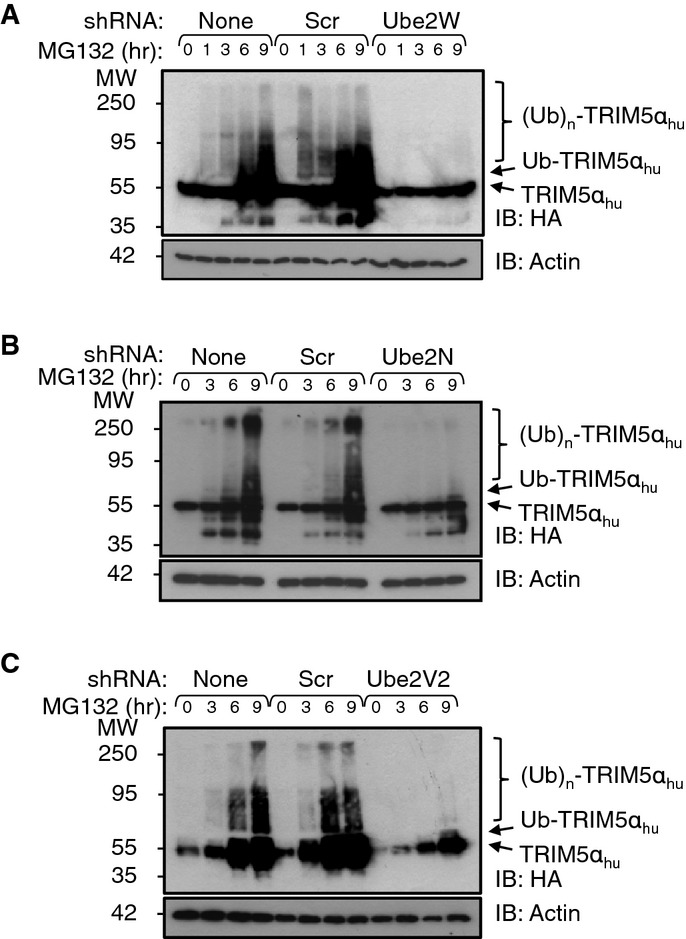
Depletion of Ube2W, Ube2N, or Ube2V2 suppresses TRIM5α ubiquitination A–C TE671 cells stably expressing HA-TRIM5α_hu_ and shRNAs targeting Ube2W (A), Ube2N (B) or Ube2V2 (C) or a scrambled control shRNA (Scr), or no shRNA (None), were treated with MG132 for the designated time intervals, and cell lysates were analyzed by IB detection of HA-TRIM5α_hu_ (HA), or β-actin (loading control). All blots are representative of at least three independent experiments.

We next tested whether the E2 enzymes required for TRIM5α_hu_ restriction of N-MLV DNA synthesis also participate in TRIM5α_hu_ ubiquitination. As shown in Fig3, single depletions of Ube2W, Ube2N, or Ube2V2 each strongly inhibited the formation of HMW TRIM5α_hu_ Ub adducts that otherwise accumulated upon proteasome inhibition. However, the resulting distributions of TRIM5α_hu_ species varied in the different cases. Ube2W depletion abrogated all detectable TRIM5α_hu_ ubiquitination (Fig3A), whereas depletion of either Ube2N or Ube2V2 prevented polyUb chain formation but did not prevent formation of a monoubiquitin (monoUb) TRIM5α_hu_ species (Ub-TRIM5α_hu_, Fig3B and C). The similar accumulation of Ub-TRIM5α_hu_ following depletion of either Ube2N or Ube2V2 is consistent with the idea that these two E2 activities function together as a single Ub E2 conjugating enzyme. Together, our observations are consistent with a model in which Ube2W mediates TRIM5α monoubiquitination and the Ube2N/Ube2V2 enzyme then extends the polyUb chain from the anchoring Ub.

### TRIM5α is modified with Lys63-linked polyUb chains in cells

**Figure 4:**
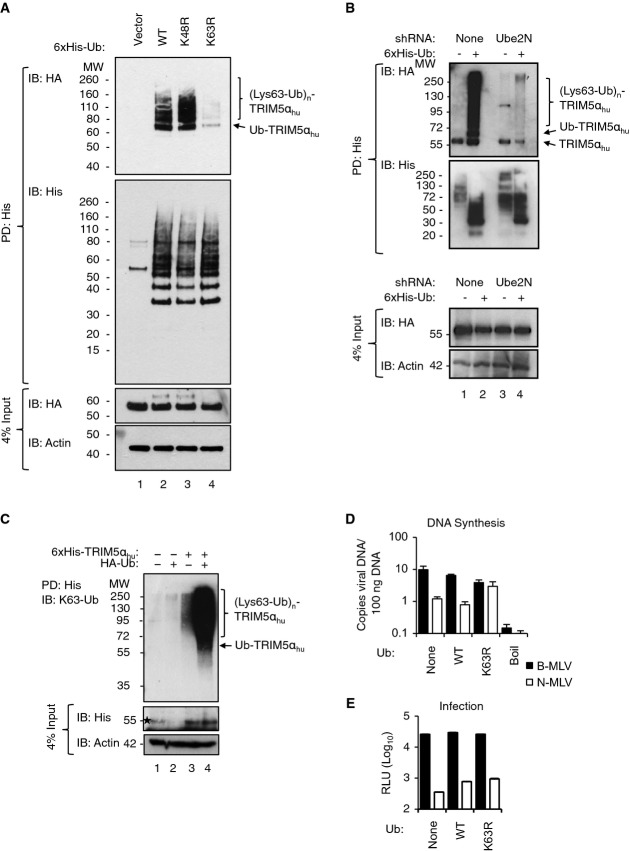
The polyUb chains on TRIM5α contain Lys63 linkages, and Lys63-linked polyUb chain formation is necessary for restriction of N-MLV DNA synthesis A (Ub)_n_-TRIM5α_hu_ synthesis is inhibited by expression of K63R but not K48R mutant Ub. IB of PD products on Ni^2+^ affinity matrices from lysates of TE671 cells expressing HA-TRIM5α_hu_ transfected with empty vector or 6×His-tagged Ub WT, Ub K48R, or Ub K63R. TRIM5α_hu_ species were detected by IB for the HA-tag (HA), and equivalent purifications of His-labeled ubiquitinated proteins for each Ub variant were confirmed by IB detection of the 6×His tag (His). Input fractions were analyzed to confirm that similar levels of TRIM5α_hu_ were present before purification.

The observations that HMW TRIM5α_hu_ species accumulate in the absence of proteasome activity and that depletion of either Ube2W or Ube2N/Ube2V2 blocks this accumulation were surprising because proteasome recruitment and protein degradation are typically mediated by Lys48 (K48)-linked polyUb chains, whereas Ube2W typically modifies proteins with monoUb and the Ube2N/Ube2V2 heterodimer typically builds Lys63-linked polyUb chains (K63-Ub) (VanDemark *et al*, 2001; Christensen *et al*, 2007; Scaglione *et al*, 2013; Tatham *et al*, 2013). We therefore characterized the nature of the polyUb linkages within the TRIM5α_hu_ HMW species by co-expressing and purifying mutant Ub proteins that either could not form K63-Ub (Ub K63R) or could not form K48-linked chains (Ub K48R). As shown in Fig4A, formation of (Ub)_n_-TRIM5α_hu_ species was normal after expression of Ub K48R, but was efficiently blocked by expression of Ub K63R, consistent with the idea that Ube2N/Ube2V2 catalyzes formation of K63-Ub on TRIM5α_hu_ (Fig4A). Importantly, Ub K63R species were present at normal levels in cell lysates as verified by detecting the 6×His tag (Fig4A, panel 2). The Ub K63R-mediated suppression of TRIM5α_hu_ HMW species was as efficient as the suppression seen upon depletion of Ube2N (Fig4B). These observations suggest that TRIM5α is specifically labeled with K63-Ub by Ube2N/Ube2V2. In further support of this hypothesis, an antibody specific for K63-linked Ub chains reacted well with affinity-purified His-TRIM5α_hu_ HMW species (Fig4C). In this case, K63-linked (Ub)_n_-TRIM5α_hu_ species were detected in the absence of exogenous Ub expression (Fig4C, lane 3) and were even more prevalent upon Ub overexpression (Fig4C, lane 4). Thus, the (Ub)_n_-TRIM5α_hu_ species contain K63 linkages.

B Ube2N depletion prevents accumulation of HMW (Ub)_n_-TRIM5α species. IB of PD products on Ni^2+^ affinity matrices from lysates of TE671 cells expressing HA-TRIM5α_hu_ and the designated combinations of 6×His-Ub and a Ube2N-specific shRNA. Note that some unmodified TRIM5α_hu_ associated non-specifically with the Ni^2+^ affinity matrix in all cases.

C Affinity-purified (Ub)_n_-TRIM5α_hu_ contains Lys63 linkages. IB of PD products from lysates of 293T cells expressing the designated combinations of 6×His-TRIM5α_hu_ and HA-Ub. Ubiquitinated products were detected with an antibody specific for Lys63-linked Ub chains (denoted K63-Ub). Input levels of 6×His-TRIM5α_hu_ (His) and β-actin (loading control) are shown below. Asterisk denotes a non-specific band in the input fraction.

D, E K63R mutant Ub expression rescues TRIM5α-restricted N-MLV DNA synthesis but not infectivity. TE671 cells that stably expressed WT or K63R 6×His-Ub-GFP, or parental TE671 cells, were transduced with either N- or B-MLV-Luc vectors (MOI ˜0.2). Copies of viral DNA at 6 h p.i. (TaqMan luciferase qPCR) (D) and relative infection at 48 h p.i. [luciferase readout, relative light units relative to calibrated light standard (Promega) (RLU)] (E) were determined in parallel samples and are plotted as read by the luminometer (GloMax, Promega) without manipulation. Boiled virus served as a negative control. Mean ± SEM of triplicates. Data information: All blots are representative of at least three independent experiments.

To test the functional importance of K63-linked polyUb modification of TRIM5α for restriction, we overexpressed Ub K63R in TE671 cells and assayed restriction of N-MLV DNA synthesis. Like Ube2N depletion (Fig1), Ub K63R overexpression restored restricted N-MLV DNA synthesis to nearly the same level as the unrestricted B-MLV control (Fig4D). Once again, viral infectivity was not substantially restored (Fig4E), as measured in a luciferase-based infectivity assay. Together, these results indicate that Ube2N/Ube2V2 adds K63-linked polyUb chains to TRIM5α and that these modifications are required for restriction of N-MLV DNA synthesis.

### The N-terminus of TRIM5α is quantitatively acetylated in cells

Ube2W has been shown to catalyze formation of a conventional peptide bond between the C-terminus of Ub and the free N-terminus of several proteins *in vitro* (Scaglione *et al*, 2013; Tatham *et al*, 2013; Vittal *et al*, 2015). However, most eukaryotic proteins are co-translationally acetylated at their N-termini (αN-acetylation), often following removal of the initiator methionine (Van Damme *et al*, 2011). αN-acetylation should block N-terminal ubiquitination because the resulting amide nitrogen is no longer nucleophilic. The TRIM5α N-terminal sequence, (M)ASGIL, should be a good substrate for αN-acetylation (Polevoda & Sherman, 2003), and TRIM5α αN-acetylation has been reported previously (Bienvenut *et al*, 2012). We confirmed this observation by analyzing C-terminally FLAG-One-*Strep* (FOS)-tagged human or rhesus macaque (rh) TRIM5α proteins expressed in human 293T cells. The exogenously expressed TRIM5α-FOS proteins were purified by *Strep*–Tactin affinity chromatography, separated by SDS–PAGE, and digested with trypsin, and the tryptic peptides were analyzed by liquid chromatography coupled with tandem mass spectrometry (LC/MS/MS). In both constructs, we identified N-terminal peptides that lacked the terminal methionine and were acetylated on the penultimate Ala residue, whereas non-acetylated or methionylated peptides were not detected in either case (Supplementary Fig S2A for human and Supplementary Fig S2B for rhesus). Hence, human and rhesus TRIM5α proteins are N-terminally acetylated in human cells, which raises the question of how they might be monoubiquitinated by Ube2W.

### Ube2W monoubiquitinates full-length TRIM5α *in vitro*

To characterize how Ube2W and Ube2N/Ube2V2 can function together with TRIM5α, we tested their activities in reconstituted systems using pure recombinant proteins. These experiments employed full-length rhesus TRIM5α proteins (TRIM5α_rh_), which are expected to utilize the same set of functionally important Ub E2 enzymes as TRIM5α_hu_ proteins because TRIM5α_rh_ efficiently restricts retroviral DNA synthesis when expressed in human cells (Stremlau *et al*, 2004). The C-terminally tagged TRIM5α_rh_-FOS was expressed in insect cells, purified by *Strep*–Tactin affinity chromatography, treated with precision protease to remove the FOS tag, and purified to homogeneity by gel filtration chromatography. Electrospray mass spectrometric analysis of the intact purified recombinant TRIM5α_rh_ protein indicated that demethionylation and αN-acetylation were complete because species representing methionylated or demethionylated and non-acetylated forms of TRIM5α_rh_ were not detected (Supplementary Fig S3). These data indicate that our recombinant TRIM5α_rh_ protein had the same N-terminal sequence and modification pattern as TRIM5α proteins expressed in human 293T cells. Ub (Pickart & Raasi, 2005), E1 (UBA1), and E2 enzymes were all expressed in *E. coli* and purified to homogeneity for use in the *in vitro* ubiquitination assays, see Materials and Methods section.

**Figure 5:**
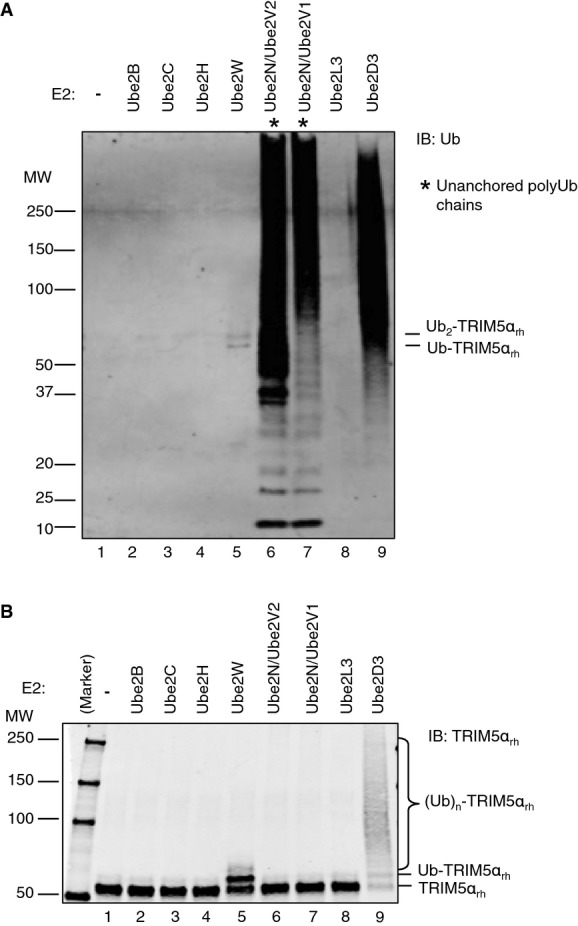
Ube2W monoubiquitnates TRIM5α *in vitro* A, B *In vitro* TRIM5α_rh_ ubiquitination reaction products analyzed by IB detection using anti-Ub (A) and anti-TRIM5α_rh_ (B) antibodies. Reactions were carried out in the presence of purified recombinant UBA1 (0.2 μM), Ub (10 μM), TRIM5α_rh_ (0.4 μM), ATP (5 mM), and the designated E2 enzyme (0.4 μM). After ATP addition, reactions were incubated for 30 min at 37°C. Reactions were stopped by addition of 2× SDS sample buffer containing 2-mercaptoethanol as a reducing agent. Unanchored polyubiquitin (polyUb) chains are indicated by an asterisk. See also Supplementary Fig S6. All blots are representative of at least three independent experiments.

To assay Ub ligase activity, TRIM5α_rh_ was incubated with UBA1, Ub, and ATP, together with the different E2 enzymes that were identified in our cellular assays (Ube2W or Ube2N/Ube2V2 or the closely related Ube2N/Ube2V1), or with one of five different control E2 enzymes (Ube2B, Ube2C, Ube2H, Ube2L3, or Ube2D3). We verified that each purified E2 was active and efficiently charged with Ub by UBA1 (Supplementary Fig S4). TRIM5α_rh_-dependent Ub ligase activity was monitored by IB, with an anti-Ub antibody used to detect formation of both unanchored and TRIM5α_rh_-anchored ubiquitination products (Fig5A), and an anti-TRIM5α_rh_ antibody used to monitor formation of TRIM5α_rh_-anchored ubiquitination products alone (Fig5B). Ube2D3 was used as a positive control in these experiments because this E2 enzyme is highly promiscuous and has previously been shown to be capable of ubiquitinating TRIM5α_rh_ *in vitro* (Yamauchi *et al*, 2008; Kim *et al*, 2011). As expected, Ube2D3 catalyzed the formation of TRIM5α_rh_-linked polyUb chains, and possibly also unanchored chains (Fig5A and B, lane 9). However, Ube2D3 lacked chain linkage specificity, because individual Ub lysine mutations could not perturb TRIM5α_rh_ autoubiquitination (Supplementary Fig S5A and Table1). Moreover, Ube2D3 does not function in TRIM5α restriction because its efficient depletion did not significantly abrogate TRIM5α restriction of N-MLV DNA synthesis or infectivity (Supplementary Fig S5B–D).

**Table 1:** TRIM5α_rh_ polyUb chain linkages produced with different E2 enzymes

E2:	None	Ube2W	Ube2N/V2	Ube2N/V2 + Ube2W	Ube2D3
Lys6	NP	NP	NP	NP	NP
Lys11	NP	NP	NP	NP	320,000
Lys27	NP	NP	NP	NP	NP
Lys29	NP	NP	NP	NP	NP
Lys33	NP	NP	NP	NP	NP
Lys48	NP	NP	NP	NP	180,000
Lys63	NP	NP	8,000	60,000	92,000
Met1	NP	NP	NP	NP	NP

TRIM5α_rh_ ubiquitination products were produced in the reactions shown in Supplementary Fig S6. Ub chain linkages were then determined by tryptic digestions of the reaction mixtures and LC/MS/MS analyses of the resulting peptides. Values represent spectral counts for any diagnostic peptides detected. The absence of diagnostic peptides is denoted NP (not present).

As shown in Fig5, the closely related Ube2N/Ube2V1 and Ube2N/Ube2V2 E2 enzymes could collaborate with TRIM5α_rh_ to synthesize polyUb chains (Fig5A, lanes 6 and 7) that were not covalently anchored to TRIM5α_rh_ (Fig5B, lanes 6 and 7). This observation agrees well with a previous report that Ube2N/Ube2V1 and TRIM5α_rh_ synthesize unanchored K63-Ub (Pertel *et al*, 2011). Ube2W was the only other assayed E2 that exhibited any observable enzymatic activity (Fig5, lane 5). In this case, however, the enzyme predominantly attached a single Ub molecule directly onto TRIM5α_rh_, and also made a small amount of TRIM5α_rh_ with two attached Ub molecules (Fig5B, lane 5). The ability of Ube2W to transfer a single Ub onto TRIM5α_rh_ *in vitro* is consistent with the pattern of ubiquitination seen in cells, where depletion of Ube2N/Ube2V2 activity led to accumulation of Ub-TRIM5α_hu_ (Fig3B and C) and depletion of Ube2W blocked all detectable TRIM5α_hu_ ubiquitination (Fig3A). Thus, Ube2W can catalyze the addition of a single Ub onto TRIM5α in cells, and this activity can be recapitulated *in vitro* using pure recombinant proteins.

### Ube2N/Ube2V2 builds Lys63 polyUb chains on Ub-TRIM5α *in vitro*

**Figure 6:**
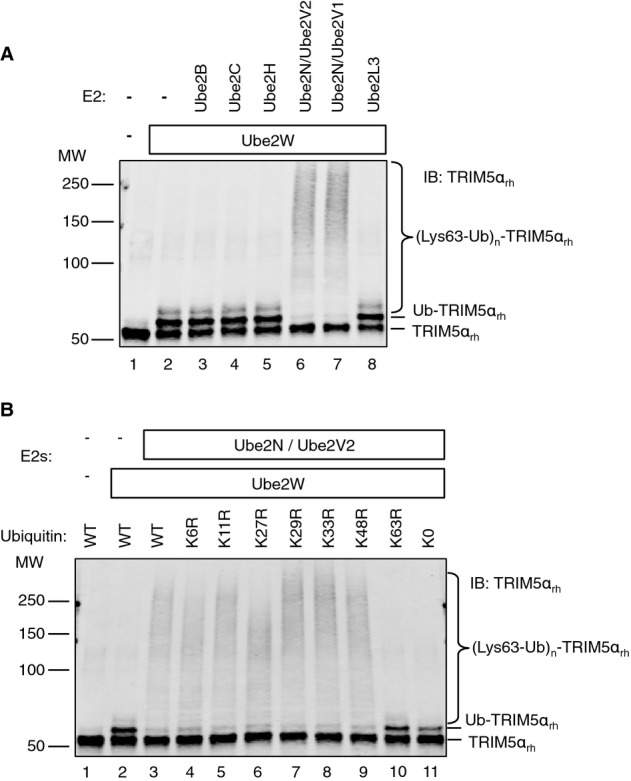
Ube2N-containing E2 enzymes build anchored Lys63-linked polyUb chains on Ub-TRIM5α TRIM5α_rh_ ubiquitination reactions used the same conditions as those shown in Fig5, but with multiple E2 enzymes. Ube2W (lanes 2–8) was used to attach anchoring monoUb molecules to TRIM5α_rh_ *in situ*, and the indicated E2 enzymes were tested for polyUb chain elongation from the anchored Ub of Ub-TRIM5α_rh_.

Previous studies have shown that Ube2N/Ube2V2 and Ube2N/Ube2V1 can extend a polyUb chain from an anchored Ub that is attached to a substrate by a different E2, such as Ube2W (Christensen *et al*, 2007; Tatham *et al*, 2013). We therefore tested whether Ube2N-containing enzymes could use Ub-TRIM5α_rh_ proteins as substrates for further ubiquitination. Ub-TRIM5α_rh_ proteins were created *in situ* by incubating Ube2W with TRIM5α_rh_ (Fig6A, lane 2), and a panel of six different E2 enzymes was tested for the ability to use these Ub-TRIM5α_rh_ proteins as substrates to create TRIM5α_rh_-anchored polyUb chains (Fig6A lanes 3–8). Ube2N/Ube2V2 and Ube2N/Ube2V1 enzymes both exhibited this activity (Fig6A, lanes 6 and 7, respectively)—note significant depletion of the band representing Ub-TRIM5α_rh_ with concomitant appearance of TRIM5α_rh_-anchored polyUb—whereas none of the control E2 enzymes tested showed any observable activity (Fig6A, lanes 3–5 and 8). As expected, the TRIM5α_rh_-linked polyUb chains synthesized by Ube2N/Ube2V2 contained K63 linkages, as analyzed both by testing the activities of single K/R Ub mutants (Fig6B) and by trypsin digestion/mass spectrometry mapping experiments (Table1). Furthermore, we observed a noticeable difference in the pattern of TRIM5α_rh_ polyUb products produced by Ube2N/Ube2V2 in the presence versus absence of Ube2W (Supplementary Fig S6A, compare lane 3 to lane 4). In the absence of Ube2W, darker staining of the IB was observed in a region representing very high MW polyUb products, which likely represent longer chains. In contrast, the presence of Ube2W led to more intense staining over a broader region of the IB, indicating an overall increase in the number of Ub linkages. This overall increase was confirmed by quantifying the overall staining intensity of ubiquitination products (lane 3 intensity was 354, lane 4 intensity was 954). The increase in K63 linkages was also supported by an eight-fold increase in mass spectrometric spectral counts (Table1).

Mutant Ub proteins with the designated Lys to Arg mutations were used to identify the residue(s) used for polyUb chain extension. K0 Ub has all seven lysine residues mutated to arginine. Data information: IBs are representative of three experiments. See also Supplementary Fig S6.

Collectively, these results are again in excellent agreement with the patterns of TRIM5α ubiquitination seen in cells, where Ube2N/Ube2V2 adds K63-Ub, but can only do this when Ube2W is present (Figs1 and 3). We therefore conclude that Ube2W and Ube2N/Ube2V2 can modify TRIM5α in a sequential reaction in which Ube2W first adds a single Ub molecule, and Ube2N/Ube2V2 then builds K63-Ub onto the anchoring Ub molecules.

### Ube2W transfers ubiquitin on to internal TRIM5α lysine residues

Mass spectrometric analyses were performed to map the site(s) of TRIM5α_rh_ autoubiquitination. In this case, the Ube2W/TRIM5α_rh_ ubiquitination reaction was performed on a preparative scale to drive the reaction to completion and to produce sufficient quantities of products for analysis. Mono- and di-ubiquitinated TRIM5α_rh_ products were purified by SDS–PAGE (Supplementary Fig S7A, lanes 4 and 5), and digested with trypsin, and ubiquitinated tryptic peptides were then identified by LC/MS/MS based upon the presence of remnant isopeptide-linked Gly-Gly dipeptides (Peng *et al*, 2003; Flick *et al*, 2004). This analysis identified sites of TRIM5α_rh_ ubiquitination on Lys residues 45 and 50 (Supplementary Fig S7B; D.E. Christensen, C. Nelson, W.I. Sundquist, unpublished observations). Lys45 and Lys50 are located within the RING domain, near the expected Ube2W binding site. As expected, peptides corresponding to the acetylated protein N-terminus were detected, and no other free or modified N-terminal peptides were detected. A peptide corresponding to K48-linked diUb was also detected in these reaction mixtures, but this product may arise owing to the high levels of ubiquitinating enzymes used in this preparative reaction because this product was not detected in other Ube2W ubiquitination reactions where lower enzyme levels were used (see Table1).

The observation that Ube2W could add isopeptide-linked monoUb modifications to internal TRIM5α_rh_ Lys residues was noteworthy because Ube2W has previously been reported to preferentially couple Ub to the free αN-termini of proteins via conventional peptide bonds (Scaglione *et al*, 2013; Tatham *et al*, 2013). We therefore tested the idea that the TRIM5α_rh_ αN-acetyl group was blocking Ube2W-mediated N-terminal ubiquitination, using a different recombinant TRIM5α_rh_ protein that had a free, unacetylated N-terminus (denoted *TRIM5α_rh_). *TRIM5α_rh_ was obtained by expressing an N-terminally tagged FOS-TRIM5α_rh_ protein in insect cells, purifying the protein by *Strep*–Tactin affinity chromatography, and removing the N-terminal affinity tag with PreScission protease to produce a protein with a free αN-terminus on the resulting non-native, N-terminal Gly-Pro extension. Ube2W added 1–3 Ub molecules onto *TRIM5α_rh_ in preparative *in vitro* ubiquitination assays, and in this case, the di-ubiquitinated products were the most prevalent species (Supplementary Fig S7A, lanes 2 and 3). Thus, Ube2W added approximately one additional Ub molecule to the non-acetylated *TRIM5α_rh_ protein than to the acetylated TRIM5α_rh_ protein. In this case, mass spectroscopic mapping experiments detected peptides that corresponded to αN-terminal ubiquitination products, as well as isopeptide-linked Ub molecules at internal Lys residues 45, 50, and less frequently to Lys residues 85, 218, 284, and 372 (see Supplementary Fig S7B, caption). These experiments demonstrate that Ube2W can conjugate Ub to the free N-terminus of the artificial *TRIM5α_rh_ construct. However, when the N-terminus is blocked by acetylation, as is the case in cells, Ub can instead be linked to internal Lys residues.

**Figure 7:**
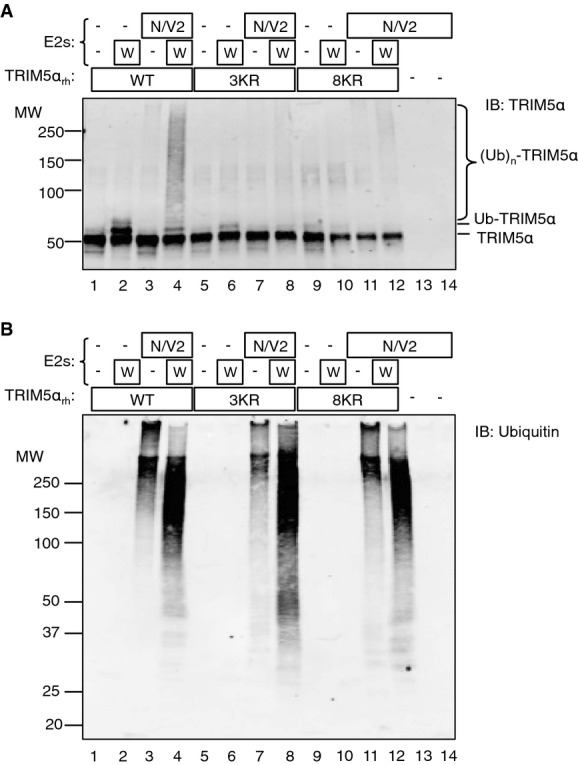
Mutation of Lys45 and Lys50 blocks TRIM5α auto-ubiquitination *in vitro* A, B *In vitro* TRIM5α_rh_ ubiquitination reaction products analyzed by IB detection using anti-TRIM5α_rh_ (A) and anti-Ub (B) antibodies and the same conditions as those shown in Fig5 but with TRIM5α_rh_ lysines mutated to arginine (3KR: K44R, K45R, K50R and 8KR: K44R, K45R, K50R, K85R, K218R, K284R, K371R, K372R; Lys44 and Lys371 were selected based on close proximity to Lys45 and Lys372). All blots are representative of at least three independent experiments.

To validate the identity of the TRIM5α_rh_ Lys residues ubiquitinated in the *in vitro* assay, we prepared mutant TRIM5α_rh_ protein with lysine to arginine substitutions at the two major sites of ubiquitination located in the RING domain (Lys45, Lys50) and at the adjacent Lys44 (TRIM5α_rh_3KR). TRIM5α_rh_3KR displayed only a weak ability to accept Ub from Ube2W (Fig7A, compare lanes 2 and 6). Moreover, the HMW TRIM5α_rh_ smear produced by incubation with Ube2W and Ube2N/Ube2V2 was much lighter for the TRIM5α_rh_ mutant presumably because there is less monoubiquitinated TRIM5α_rh_ to act as a substrate for polyubiquitination (Fig7A, compare lanes 4 and 8). Although the K44R, K45R, and K50R mutations are found within the RING domain which is responsible for E2 binding, detection of Ub on the IB revealed that both TRIM5α_rh_ mutants produced unanchored HMW Ub species on incubation with Ube2N/Ube2V2 at similar levels to WT TRIM5α_rh_, suggesting the mutations did not negatively affect E2 binding (Fig7B, compare lanes 3, 7, and 11). Therefore, while mutation of target lysine residues reduces the specific auto-ubiquitination of TRIM5α_rh_ *in vitro*, it does not prevent formation of Ub chains. Furthermore, these results confirm our observation that TRIM5α_rh_ residues Lys45 and Lys50 are the primary sites of Ub attachment by Ube2W *in vitro* and that other Lys residues are only weakly ubiquitinated by Ube2W, as evidenced by the weak band corresponding to mono-ubiquitination of TRIM5α_rh_3KR (which is then lost on addition of Ube2N/Ube2V2). Interestingly, Ube2W appears to enhance the polyubiquitination activity of Ube2N/Ube2V2 in a TRIM5α_rh_-dependent manner (Fig7B, compare lanes 4, 8, 12, and 14). This enhanced polyubiquitination activity by Ube2W is likely the result of aberrant transfer of Ub to the Ube2W N-terminus or lysines, as is observed in the E2 charging assay (Supplementary Fig S4B, lane 2, band ‡), which can then function as the anchoring Ub for polyUb synthesis by Ube2N/Ube2V2.

We then asked whether mutation at these specific lysine residues was able to similarly diminish TRIM5α ubiquitination in cells. As before, we used TRIM5α_hu_ restriction assays to readout restriction of infection and viral DNA synthesis as a measure of ubiquitination and proteasome recruitment. We prepared a TRIM5α_hu_ mutant construct (TRIM5α_hu_3KR), with an authentic N-terminus, bearing lysine to arginine substitutions at positions 44, 45, and 50 and tested its ability to restrict N-MLV infection and DNA synthesis upon expression in feline CRFK cells. Interestingly, TRIM5α_hu_3KR was able to restrict N-MLV DNA synthesis and infection as effectively as WT TRIM5α_hu_ (Supplementary Fig S8A and B). Treatment of TRIM5α_hu_-expressing CrFK cells with MG132 also revealed robust HMW modification of the TRIM5α_hu_ mutant (Supplementary Fig S8C), suggesting that additional TRIM5α lysine residues are able to accept Ub in cells. In support of this, we could specifically purify ubiquitinated species of TRIM5α_hu_3KR from cells, at levels comparable to WT protein (Supplementary Fig S8D). Therefore, mutation of these three lysine residues was unable to preclude TRIM5α_hu_ ubiquitination in cells, suggesting that ubiquitination of TRIM5_hu_ at positions additional to these is able to support TRIM5α-mediated inhibition of viral replication in cells.

We therefore examined a second pair of TRIM5α mutants. TRIM5α_rh_8KR (K44R, K45R, K50R, K85R, K218R, K284R, K371R, K372R) is mutated at all of the Lys residues found to be ubiquitinated by MS analysis as well as at lysine residues 44 and 371, selected for mutation based on close proximity to lysine residues 45 and 372. *In vitro*, TRIM5α_rh_8KR was very substantially impaired in auto-ubiquitination with Ube2W (Fig7A, compare lanes 2 and 10, and 4 and 12). Importantly, as for the WT protein, the small amount of monoubiquitination seen on TRIM5α_rh_8KR (Fig7A, lanes 9 and 10) was lost on addition of Ube2N/Ube2V2 and converted a faint HMW smear, consistent with polyubiquitination of the small amount of monoubiquitinated TRIM5α_rh_8KR. We also prepared an equivalent mutant in TRIM5α_hu_ (TRIM5α_hu_7KR). This protein has lysine to arginine substitutions at positions 44, 45, 50, 84, 282, 367, 368. Note that TRIM5α_rh_ K218 is not conserved in the human orthologue, and TRIM5α_rh_ K85, K284, K371, and K372 are equivalent to TRIM5α_hu_ residues K84, K282, K367, and K368, respectively. We did not expect TRIM5α_hu_7KR to restrict infection when expressed in cells because TRIM5α_hu_ residues Lys367 and Lys368 are located in the surface-exposed PRYSPRY loop 5 (Supplementary Fig S8E) (Biris *et al*, 2012), and their simultaneous mutation can impair restriction activity by decreasing binding to N-MLV capsid protein (Sebastian *et al*, 2009). Consistent with this expectation, TRIM5α_hu_7KR was unable to restrict either N-MLV DNA synthesis or infection (Supplementary Fig S8A and B). However, TRIM5α_hu_7KR still produced HMW species upon MG132 treatment (Supplementary Fig S8C). Similarly, ubiquitinated TRIM5α_hu_7KR species could be purified from cells, at levels similar to WT protein, even in the absence of proteasome inhibitor (Supplementary Fig S8D). We conclude that TRIM5α_hu_7KR is probably inactive owing to mutations in the PRYSPRY domain. Nevertheless, this mutant reveals that even when all of the identified *in vitro* ubiquitination sites are mutated, TRIM5α_hu_7KR can be ubiquitinated at additional sites in cells.

## Discussion

In this study, we demonstrate that the anti-retroviral restriction factor TRIM5α is labeled with Ub in cells in a Ube2W-, Ube2N-, UbeV2-, and K63-Ub-dependent manner. Depletion of Ube2W abolished all conjugation of Ub to cellular TRIM5α, whereas depletion of Ube2N or Ube2V2 abolished poly- but not monoubiquitination (Fig3). Reconstitution of ubiquitination reactions *in vitro* using purified full-length TRIM5α revealed that TRIM5α efficiently catalyzed auto-monoubiquitination using Ube2W (Fig5) and that this modification was converted into anchored K63-Ub by addition of Ube2N/Ube2V2 (Fig6). Our data add TRIM5α to the growing list of E3 Ub ligases shown to catalyze Ube2W-dependent monoubiquitination *in vitro* (Christensen *et al*, 2007; Alpi *et al*, 2008; Scaglione *et al*, 2011; Zhang *et al*, 2011; Tatham *et al*, 2013; Vittal *et al*, 2015). The cooperation between Ube2W and Ube2N/Ube2V2 has also been observed *in vitro* (Christensen *et al*, 2007; Tatham *et al*, 2013). To the best of our knowledge, however, TRIM5α represents the first instance in which the sequential ubiquitination mechanism has been dissected in cells using assays for protein function.

In experiments where the relevant E2 enzyme was depleted, or Ub K63R was expressed, we observed that TRIM5α ubiquitination activity correlated with its restriction of retroviral DNA synthesis by reverse transcription, but not with its inhibition of viral infection. Interestingly, inhibition of proteasomes with small molecules (Anderson *et al*, 2006; Wu *et al*, 2013) also rescues restricted reverse transcription but not infection. Our data therefore suggest that the role of TRIM5α ubiquitination is not to prevent viral infection per se, but to block viral reverse transcription, through a mechanism that also requires proteasome activity. This model is consistent with those in which viral infectivity is suppressed when TRIM5α forms a complementary super lattice on the viral capsid and prevents progression to proper uncoating and nuclear entry (Stremlau *et al*, 2006; Wu *et al*, 2006; Ganser-Pornillos *et al*, 2011); however, our data cannot formally exclude a role for viral capsid ubiquitination in the TRIM5α restriction mechanism.

Pertel *et al* (2011) have postulated that the role of TRIM5α Ub ligase activity is to stimulate an innate immune response through the synthesis of unanchored K63-Ub and that this activity is necessary for TRIM5α to restrict viral infection. While we do not find a requirement for ubiquitination activity in TRIM5α restriction of viral infection (Figs1 and 4), our data are compatible with a model in which TRIM5α synthesizes unanchored polyUb, as we too observed unanchored polyUb chain synthesis *in vitro* using recombinant TRIM5α and either Ube2N/Ube2V2 or Ube2N/Ube2V1 (Fig5). However, our findings that: (i) Ube2W was absolutely required for restriction of viral DNA synthesis, and not infection, and (ii) that Ube2W causes the K63-Ub to be anchored to TRIM5α itself suggest a novel mechanism whereby anchored, and not free, K63-Ub is the necessary signal to trigger downstream effector mechanisms that result in a block to viral reverse transcription in a proteasome-dependent manner. We also note that addition of Ube2W to reactions between TRIM5α and Ube2N/Ube2V2 results in a greater abundance of polyUb, and the produced chains are also shorter (Supplementary Fig S6). How K63-Ub could underlie proteasome recruitment remains unclear, although we envisage either a mechanism of Ub chain branching whereby the introduction of additional Ub chain linkages confers additional function (Nakasone *et al*, 2013) or via the recruitment of K63-Ub-specific Ub binding domains that facilitate transfer of ubiquitinated TRIM5α, bound to a viral capsid, to the proteasome. A potential role for autophagy in TRIM5α antiviral activity was recently described (Mandell *et al*, 2014), lending support to the notion that additional cofactors of TRIM5α remain to be discovered. Moreover, an alternative autophagic degradation pathway could explain how K63-Ub induces degradation, with the effects of proteasome inhibition explained by concomitant disturbances in Ub homeostasis.

Mass spectrometry mapping experiments have helped us understand how Ube2W mediates TRIM5α polyUb “anchoring” to TRIM5α. Analyses of trypsin-digested TRIM5α expressed in cells revealed that the N-terminus of TRIM5α is quantitatively acetylated—a modification that blocks N-terminal ubiquitination. *In vitro* ubiquitination of αN-acetylated-TRIM5α followed by mass spectrometry analyses demonstrated that Ube2W can catalyze monoubiquitination of wild-type TRIM5α at multiple lysine residues, predominantly Lys45 and Lys50 in the TRIM5α RING domain. In lower abundance, we also detected ubiquitination of lysines 85, 218, 284, and 372. While αN-acetylated N-terminal peptides were detected in our mass spectrometry experiments, no N-terminally ubiquitinated peptides were detected (Supplementary Fig S2). Ube2W is reported to target protein N-termini preferentially (Scaglione *et al*, 2013; Tatham *et al*, 2013; Vittal *et al*, 2015) and, indeed, we found that Ube2W could add Ub to the N-terminus when this site was (artificially) unblocked. Thus, our data show that although Ube2W can target protein N-termini, it can also target internal lysine residues, as is the case for native, WT TRIM5α_rh_.

We were able to validate the importance of these six (and two adjacent) Lys residues *in vitro*, as TRIM5α_rh_ mutated at eight lysine residues was a poor substrate for Ube2W-mediated monoubiquitination. However, mutation of the corresponding seven lysine residues in TRIM5α_hu_ did not substantially diminish TRIM5α_hu_ ubiquitination in cells, suggesting that additional lysine residues can be ubiquitinated, at least when the primary targets are mutated. Similarly, while TRIM5α_rh_ mutated at lysine residues 44, 45, and 50 was also impaired for *in vitro* ubiquitination with Ube2W, these mutations did not diminish TRIM5α_hu_ ubiquitination or restriction activity in cells. One possible explanation for this discrepancy is that TRIM5α is not itself the functional target of the K63-Ub that is essential for restriction of viral DNA synthesis. However, the loss of ubiquitinated TRIM5α species after either E2 enzyme depletion, or Ub K63R expression in cells, correlates with the loss of restriction of viral DNA synthesis. This observation supports the notion that TRIM5α itself is the functional target of ubiquitination (Figs1, 3 and 4). Another possible explanation is that we have failed to mutate all of the possible target lysines in TRIM5α. Our mass spectrometry demonstrated that TRIM5α ubiquitination is not highly specific. *In vitro*, the RING domain lysines at positions 45 and 50 are the major targets for autoubiquitination, possibly because these lysines are the closest to the E2 binding site in the RING domain. However, conformations adopted by TRIM5α assemblies in cells, perhaps aided by additional cofactors, might orientate the protein such that additional lysines become favored ubiquitination targets. We observed a hint of Ube2W-catalyzed monoubiquitinated TRIM5α_rh_ bearing three or eight lysine–arginine substitutions *in vitro*, which was lost on Ube2N/Ube2V2 addition (Fig7A, compare lanes 6 and 7), suggesting that in the absence of these amine groups, TRIM5α might ubiquitinate lysines more distant to the E2 binding site, albeit less efficiently in our *in vitro* reactions. Finally, it is possible that other E3 ligases functionally ubiquitinate TRIM5α via the E2 enzymes identified here. However, our observation that TRIM5α is able to autoubiquitinate *in vitro* using these enzymes makes TRIM5α itself the most likely E3 candidate for TRIM5α-dependent restriction.

Although our data do not prove that TRIM5α is the functional target for Ub-dependent restriction, we favor a model in which TRIM5α autoubiquitination is necessary for subsequent inhibition of viral DNA synthesis. In principle, TRIM5α-anchored Ub molecules offer the advantage that they would presumably remain associated with the incoming viral capsid and thus could “mark” that capsid for inactivation. Furthermore, redundancy of target lysines for ubiquitination is consistent with a protein whose effective function depends on combating rapidly evolving pathogens by evolving changes in antiviral specificity. Plasticity in the lysines used as ubiquitination targets would reduce the likelihood that TRIM5α sequence changes would impact its ability to autoubiquitinate. Redundancy of target lysines may therefore be an advantageous, evolved property of the TRIM5α protein.

In summary, we have identified a novel series of reactions catalyzed by TRIM5α that result in the anchoring of K63-Ub to TRIM5α itself. Perturbation of the enzymatic activities involved in the formation of this modification prevents TRIM5α from restricting retroviral infection prior to viral reverse transcription. This hitherto unidentified mechanism appears to lie in the same pathway as proteasome-dependent disassembly of retroviral capsids. Also, the observed K63-Ub specificity suggests that a relationship may exist between TRIM5α restriction of retroviral reverse transcription and stimulation of innate immune signaling cascades. Our study highlights the value of combining *in vitro* assays for ubiquitination with cellular assays for Ub ligase function. The ability to measure TRIM5α ubiquitination activity functionally in cells, using different steps in the retroviral lifecycle as the readout, makes this an attractive approach for further studies of TRIM protein-mediated ubiquitination and for analyzing the contribution of K63-Ub to proteasome recruitment and innate immune signaling.

## Materials and Methods

### Cells, viral vectors, chemicals and antibodies

HeLa, TE671, 293T, and CrFK cells were obtained from the American Type Culture Collection and grown in DMEM (Invitrogen) with 10% FCS (Biosera) at 37°C in 5% CO_2_, (293T in DMEM with 15% FCS, at 37°C in 10% CO_2_) and were tested negative for mycoplasma (Lonza). All media contained 100 U/ml penicillin and 100 μg/ml streptomycin. VSV-G pseudotyped viral particles were generated by three-plasmid transfection of 293T with Fugene-6 (Promega), using 1 μg Gag-Pol expression plasmid, 1 μg VSV-G expression plasmid pMD2.G (GenScript), and 1.5 μg genome vector. Plasmids used were as follows: pCMVi (MoMLV Gag-Pol), pCIG3-N (N-tropic MLV Gag-Pol), pCIG3-B (B-tropic MLV Gag-Pol), and pCNCG (MLV-GFP genome). Human TRIM5α and TRAF6 were cloned from human cell cDNA and inserted into the retroviral expression vector EXN (a kind gift from Paul Bieniasz), which express N-terminally HA-tagged proteins. The HA-tag was deleted by overlap PCR in order to express C-terminally HA-tagged proteins. TRIM5α mutants were generated by site-directed mutagenesis (Stratagene). Ubiquitin (Ub) proteins were expressed from either pMT123 (HA-tagged Ub) or pMT107 (6×His-tagged Ub), which encode a multimeric precursor molecule comprised of eight consecutive Ub units downstream of the N-terminal tag (Treier *et al*, 1994), pcDNA3.1-Kozak-6×His-UbGFP, or pHR’-SIN-6×His-UbGFP-WT/-K48R/-K63R. In the latter two constructs, Ub and GFP are post-translationally cleaved producing Ub monomers and GFP. MG132 (Calbiochem) was used at 2 μg/ml. Antibodies used in this study were as follows: rat monoclonal to HA-tag (3F10, Roche, 1:5,000), rabbit polyclonal to Ube2L3 (A-640, Boston Biochem, 1:1,000), rabbit polyclonal to Ube2N (AB10025, Millipore, 1:1,000), mouse monoclonal to β-actin (AC-15, Abcam, 1:40,000), mouse monoclonal to His-tag (H8, Millipore, 1:1,000, or Penta-His 34660, Qiagen, 1:1,000), mouse monoclonal to TRIM5α_rh_ (5D5-1-1, distributed by the NIH AIDS Reagent Repository), mouse monoclonal to Ub (P4D1, Santa Cruz Biotechnology), and rabbit monoclonal to Ub, Lys63-specific clone Apu3 (Merck Millipore). HRP-conjugated secondary antibodies (mouse, rabbit) were from GE Healthcare and used at 1:5,000. HRP was detected with ECL (GE Healthcare) or RapidStep ECL (Calbiochem). A complete list of expression plasmids, antibodies, siRNAs, oligonucleotides, and real-time qPCR probes used in this study is provided in Supplementary Table S1.

### RNAi-mediated E2 ubiquitin-conjugating enzyme library screen

The siRNA library screen for 38 human E2 Ub-conjugating enzymes constituted 38 siGENOME SMARTpools (Dharmacon), one pool per well in a 96-well plate. Pools were resuspended in Optimem and Oligofectamine. 8 × 10^3^ HeLa cells were added to each pool, with siRNA concentrations of 30 nM in final volumes of 100 μl. The cells were incubated at 37°C, washed after 24 h, and incubated for a further 24 h. Cells were then infected with N-tropic or B-tropic MLV-GFP vectors (MOI ∼0.2) in a final volume of 200 μl.

### shRNA-mediated depletion of Ube2W, Ube2N, and Ube2V2

shRNA-encoding oligonucleotides were designed, annealed, and ligated into pSIREN Retro Q (Clontech) according to manufacturer’s instructions (http://www.clontech.com/GB/Support/Online_Tools). The 19mer target sequences are listed in Supplementary Table S1. The shRNA sequence designed to deplete Ube2V2 corresponded to the most effective 19mer target sequence from a screen of four independent siRNA oligonucleotides. The Ube2N 19mer sequence was previously described (Duncan *et al*, 2006). The Ube2W 19mer sequence targeting the 3′UTR was designed according to Clontech instructions. The Ube2V1 22mer sequence was previously described (Pertel *et al*, 2011). TE671 cells were transduced at an MOI ∼1 and selected in 2.5 μg/ml puromycin (Fletcher *et al*, 2010).

### Infectivity and quantitative polymerase chain reaction (qPCR) assays

10^5^ TE671 cells per well were seeded in 12-well plates and transduced 24 h later (MOI ∼0.2) in quadruplicate with RQ1 DNase-treated (30 U/ml, 2 h, 37°C, in buffer containing 400 mM Tris (pH 8.0), 100 mM MgSO_4_, and 10 mM CaCl_2_) N-MLV or B-MLV GFP-expressing viral vectors, in the presence of 8 μg/ml Polybrene. Heat-treated viral supernatants (95°C, 5 min) served as controls for plasmid contamination. 6 h p.i., duplicate wells were washed in PBS, trypsinized, pelleted, washed again in PBS, pelleted, and stored at −80°C. Total DNA was extracted using QIAamp DNA Mini Kit (Qiagen), eluting DNA in 50 μl water. Parallel duplicate wells were scored for GFP expression by flow cytometry (BD Accuri C6). Concentration of each DNA sample was quantified using a NanoDrop spectrophotometer ND-1000 (Thermo Fisher Scientific). TaqMan qPCR reactions contained 5 μl of eluted DNA, 300 nM forward and reverse primers (listed in Supplementary Table S1), 150 nM probe, 20 μg salmon sperm DNA, and 1× TaqMan Universal Master Mix II (Life Technologies). Standard curves generated from GFP cDNA plasmids were created in duplicate for each qPCR assay. Cycle parameters for GFP gene detection were 50°C for 2 min and 95°C for 10 min, followed by 40 cycles of 95°C for 15 s, and 60°C for 1 min using a Prism 7000 thermocycler (Applied Biosystems). SYBR Green qPCR assays were used to detect transcript abundance of E2s, using 1 μM primers as listed in Supplementary Table S1, and ∼200 ng cDNA. Cycle parameters were 5 min at 95°C, followed by 40 cycles of 95°C for 10 s and 60°C for 30 s. Standard curves generated from E2 cDNA plasmids were created in duplicate for each qPCR assay. The specificity of E2-specific primer pairs was assessed in control PCRs (Supplementary Fig S1).

### cDNA synthesis for quantitation of Ube2W, Ube2V2, and Ube2V1 depletion

Total RNA was purified using the RNeasy RNA Extraction Kit according to manufacturer’s instructions (Qiagen). 1 μg RNA was mixed with 42 ng oligo-dT primer and 0.8 mM dNTPs, heated at 65°C for 5 min, and then chilled on ice. The mixture was then supplemented to a final concentration of 250 mM Tris (pH 8.3), 375 mM KCl, 15 mM MgCl_2_, and 10 mM DTT and incubated at 42°C for 2 min. 200 units of SuperScriptTM II reverse transcriptase were then added and the mixture incubated at 42°C for 50 min. The reaction was inactivated by heating at 70°C for 15 min. RNA was degraded by addition of two units *E. coli* RNase H (Life Technologies) and incubated at 37°C for 20 min. cDNA was stored at −20°C.

### His-tag affinity purifications

To affinity-purify His-tagged proteins (termed “pull down” purifications), sub-confluent 10-cm^2^ dishes of 293T or TE671 cells were transfected with pcDNA-6×HisTRIM5α (expressing 6×His-TRIM5α), pMT107 (expressing 6×His-Ub), pMT123 (expressing HA-Ub), or pcDNA3.1-Kozak-6×His-UbGFP (expressing wild-type, K48R or K63R 6×His-Ub) with 12 μl Fugene-6 (Roche). 48 h post-transfection, cells were washed in 10 ml PBS, resuspended in 1 ml ice-cold PBS, centrifuged, and lysed in 1 ml 6 M GuHCl, 0.1 M Na_2_HPO_4_/NaH_2_PO_4_ (pH 8), and 10 mM imidazole (pH 8). Lysates were sonicated for 15 s and rotated for 3 h at room temperature with 50 μl equilibrated NiNTA agarose (Qiagen). The agarose matrix was washed twice with 1 ml lysis buffer, twice with 1 ml 3:1 wash buffer:lysis buffer, once with 1 ml wash buffer (25 mM Tris, 20 mM imidazole, pH 6.8), resuspended in 2× SDS sample buffer supplemented with 300 mM imidazole to elute bound His-tagged proteins, and 10% β-mercaptoethanol as a reducing agent, and heated for 10 min at 95°C prior to SDS–PAGE.

### *E. coli* expression vectors

Genes encoding the following E2 enzymes: Ube2B, Ube2C, Ube2D3, Ube2H, Ube2L3, Ube2N, Ube2V1, Ube2V2, and Ube2W, were amplified in PCRs to create DNA fragments that contained the E2 genes flanked by NheI and XhoI restriction sites (see Supplementary Table S1 for details). These DNA fragments were ligated into a pET11 vector modified to express proteins in frame with an N-terminal 6×His tag followed by a precision protease cleavage site. The resulting vectors were used to express recombinant E2 enzymes in *E. coli* and are designated pET11His-Ube2B (WISP11-266), pET11His-Ube2C (WISP11-267), pET11His-Ube2D3 (WISP14-34), pET11His-Ube2H (WISP11-270), pET11His-Ube2L3 (WISP14-35), pET11His-Ube2N (WISP11-269), pET11His-Ube2V1 (WISP14-36), pET11His-Ube2V2 (WISP11-272), and pET11His-Ube2W (WISP14-37).

The plasmid for expression of Ub, pET15b-Ubiquitin (WISP11-276), was a generous gift from Rachel Klevit (University of Washington). This plasmid was used as a template for site-directed mutagenesis to create vectors that expressed Ub proteins with each possible single Lys to Arg mutation as well as a Ub protein with all seven Lys residues mutated to Arg. These plasmids are designated: pET15b-Ub K6R (WISP11-277), pET15b-Ub K11R (WISP14-38), pET15b-Ub K27R (WISP14-39), pET15b-Ub K29R (WISP14-40), pET15b-Ub K33R (WISP14-41), pET15b-Ub K48R (WISP11-278), pET15b-Ub K63R (WISP11-279), and pET15b-Ub K0 (WISP14-42). The plasmid for expression of human UBA1, pET21-UBA1, was a generous gift from Cynthia Wolberger (Johns Hopkins University).

### Expression and purification of recombinant proteins

Recombinant Ub, UBA1, and E2 proteins were expressed in Rosetta(DE3) pLysS bacteria (EMD Millipore) grown in LB media and were purified to homogeneity. Briefly, human UBA1, Ube2B, Ube2C, Ube2D3, Ube2H, Ube2L3, Ube2N, Ube2V1, Ube2V2, and Ube2W were purified by affinity chromatography on Ni-NTA agarose (Qiagen) and eluted with 0.5 M imidazole in buffer containing 50 mM HEPES (pH 7.0) and 300 mM NaCl. The eluted E2 proteins were treated further with precision protease, and the solutions were incubated at 4°C overnight to remove the 6×His tags (leaving non-native Gly-Pro dipeptides at the N-termini). The UBA1 and E2 enzymes were then purified further by size exclusion chromato-graphy in 50 mM HEPES pH 7.2 and 100 mM NaCl. Ub was purified as described previously (Pickart & Raasi, 2005).

Pure rhesus TRIM5α (TRIM5α_rh_) protein was a generous gift from Vish Chandrasekaran (Li *et al*, manuscript in preparation). Briefly, Flag-One-Strep (FOS)-tagged TRIM5α_rh_ was expressed from a baculoviral vector in SF21 insect cells, purified by affinity chromatography on streptactin agarose, treated with precision protease to remove the FOS tag, and then purified to homogeneity by anion and gel filtration chromatography. The protein corresponds to wild-type TRIM5α_rh_ except that it has a non-native LEVLFQ peptide at the C-terminus.

### Ubiquitination activity assays

TRIM5α_rh_ ubiquitination assays were carried out in 30 μl reaction mixtures that contained 0.4 μM TRIM5α_rh_, 0.4 μM concentrations of the specified E2 enzyme(s), 10 μM Ub, and 0.2 μM UBA1. ATP and MgCl_2_ were added to final concentrations of 5 and 10 mM, respectively, and the reaction mixtures were incubated for 30 min at 37°C. TRIM5α_rh_ reaction products were analyzed by 4–15% SDS–PAGE gradient gels (Bio-Rad) and Western blotting. For immunoblotting, proteins were separated by SDS–PAGE, transferred to nitrocellulose membranes (Bio-Rad) in Tris-glycine–10% methanol buffer, blocked in 2% milk–Tris-buffered saline (20 min), and incubated (16 h, 4°C) with the murine monoclonal anti-TRIM5α_rh_ antibody (5D5-1-1) (1:1,000 dilution into 2% non-fat milk–Tris-buffered saline plus 0.1% Tween 20), followed by incubation with an Alexa 680 nm-labeled secondary anti-mouse antibody (Molecular Probes; 1:10,000 dilution). Blots were visualized by using an Odyssey infrared imaging system (Li-Cor, Inc.)

### Mass spectrometry

#### ESI/MS analysis of TRIM5α as an intact protein

Protein samples were purified using a C18 Ziptip™ (Millipore) to remove salts and other small molecule contaminants. The protein was loaded onto a Ziptip™ in 5% acetonitrile and then washed with 200–500 μl of 5% acetonitrile with 1% formic acid. Proteins were eluted from the Ziptip™ and collected in a pre-rinsed microcentrifuge tube with three consecutive 0.75-μl aliquots of 60% acetonitrile with 1% formic acid, then one 1-μl aliquot of 98% acetonitrile with 2% formic acid was added, and then 1 μl of 5% formic acid was added prior to ESI/MS analysis.

ESI/MS analysis of intact TRIM5α was performed using a Q-ToF-2 mass spectrometer (Waters). 3 μl of eluent was infused into the instrument with using a nanochip autosampler (Triversa NanoMate™, Advion) with nanoflow static infusion at a rate of ∼100 nl/min. Electrospray ionization was used in the positive-ion mode, but with the ion-source spray voltage of the Q-ToF-2 instrument set to zero. Ionization spray voltage was applied with the Triversa NanoMate at 1.51 kV at the nozzle of the nanochip device. Spectra were acquired with a cone voltage of 45 eV, and the instrument was scanned from 600 to 1,400 m/z in 2 s. Scans were accumulated for about 1 min. Spectra were combined, and multiply-charged molecular ions were deconvoluted into a molecular-mass spectrum processed into the average-isotope neutral molecular weight using MaxEnt™ software (Waters).

#### LC/MS/MS analysis of TRIM5α and ubiquitin

TRIM5α was analyzed by LC/MS/MS following tryptic digest of gel bands and also from solution samples. Peptides were analyzed using a nano-LC/MS/MS system comprised of a nano-LC pump (2D-ultra, Eksigent) and a LTQ-FT mass spectrometer (ThermoElectron Corporation, San Jose, CA). The LTQ-FT is a hybrid mass spectrometer in which the linear-ion trap was used for MS/MS sequencing of peptides (collision-induced dissociation (CID) fragmentation with helium collision gas), and the Fourier transform ion-cyclotron resonance (FT-ICR) part of the instrument was used to acquire MS data with high mass accuracy and high resolution. All TRIM5α peptides identified were within 3 ppm mass error compared to theoretical values, but most peptides were within 2 ppm mass error. The LTQ-FT was equipped with a nanospray ion source (ThermoElectron) at 2.3-kV spray voltage. The LTQ-FT mass spectrometer was operated in data-dependent acquisition mode controlled by Xcalibur 1.4 software, in which the “top 10” most intense peaks observed in an FT primary scan (i.e., MS survey spectrum) are subsequently trapped for MS/MS fragmentation and analysis in the LTQ linear-ion trap part of the instrument. Spectra in the FT-ICR were acquired from *m*/*z* 350 to 1,400 at 50,000 resolution at a rate of about three full scans per second. The LTQ linear-ion trap was operated with the following parameters: precursor activation time 30 ms and activation *Q* at 0.25; collision energy was set at 35%; dynamic exclusion width was set at low mass of 0.1 Da and high mass at 2.1 Da with one repeat count and duration of 10 s.

##### Nano-LC

Approximately 5–20 fmoles of tryptic digest was dissolved in 5% acetonitrile with 0.1% formic acid and injected onto a C18 nanobore LC column for nano-LC/MS/MS and identification of peptides. The nanobore column was packed and assembled homemade [C18 (Atlantis, Waters Corp); 3-μm particle; column: 75 μm ID × 100 mm length; unpacked column from New Objective]. A linear gradient LC profile was used to separate and elute peptides with a constant total flow rate of 350 nl/min. The gradient consisted of 5–80% solvent B in 78 min (solvent B: 80% acetonitrile with 0.1% formic acid; solvent A: 5% acetonitrile with 0.1% formic acid).

##### In-gel digest of proteins

SDS–PAGE gel slices were destained in 50% methanol, sliced into small pieces, and dehydrated in acetonitrile, and then the acetonitrile removed prior to digestion. The gel pieces were digested with TPCK-modified sequencing grade trypsin (Promega). 10 μl of trypsin (i.e. 20 ng/μl in 50 mM ammonium bicarbonate) was added and 20 more μl of 50 mM ammonium bicarbonate was added to the gel pieces. Digestion was allowed to continue for 2 h (at 37°C), and then two more aliquots of 20 μl of trypsin was added at 2-h intervals and then allow to continue digesting overnight. This relatively aggressive digest protocol was needed to improve the yield of peptides from Ub and TRIM5α proteins. The digestion was quenched by the addition of 20 μl 1% formic acid. This solution was allowed to stand and peptides that dissolved in the 1% formic solution were extracted and collected. Further extraction of peptides from the gel material was performed twice by the addition of 50% acetonitrile with 1% formic acid and sonicated at 37°C for 20 min, and these solutions were also collected and combined. A final complete dehydration of the gel pieces was accomplished by addition of 20 μl of 100% acetonitrile and incubation at 37°C for 20 min. The combined supernatant solutions of extracted peptides were combined and dried in a vacuum centrifuge (Speed-Vac). The peptides were reconstituted in 100 μl of 5% acetonitrile with 0.1% formic acid for LC/MS/MS analysis. After further dilution, 2% of the total digest volume was injected on the nano-LC column for LC/MS/MS analysis in a 5 μl volume.

##### Digests of proteins in solution

A 15-μl aliquot of ∼1–10 pmoles of protein in solution was digested with trypsin. Proteins in solution were digested with TPCK-modified trypsin (Promega) following the trypsin protocol outlined above. 6% of the total digest volume was injected on the nano-LC column for LC/MS/MS analysis in a 5 μl volume. All solution samples within a set of samples were analyzed identically and in a quantitative manner (i.e. sample volume, digest protocol, injection amount, and LC/MS/MS analysis).

#### Protein and peptide identifications

##### Database searches

All identified proteins and peptides from protein digest samples were assigned from protein database searches of the LC/MS/MS data, using in-house processing with MASCOT search engine (in-house licensed, ver. 2.2.7, Matrix Science, Inc.). Mascot searching was performed from an in-house computer, in which NCBI and “custom” protein databases were searched. Searches were also performed using Proteome Discoverer (Thermo, version 1.4), in which Sequest and Mascot searched were conducted. Peptides were searched assuming the following criteria:

1. Trypsin-specific cut sites, allowing for two missed cleavages.
2. Accurate mass measurement of peptide molecular ions by FTMS with search window 5 ppm (but peptides typically will have < 2 ppm mass error). Molecular ions with +1, +2, or +3 charge states were considered.
3. Peptide sequence information from MS/MS; CID fragmentation of the parent ion of each peptide was obtained in the linear-ion trap region of the LTQ-FT instrument. 0.5 Da mass error tolerance was allowed for peptide fragment ion masses in the search (but MS/MS fragment ions typically have errors < 0.3 Da).
4. Mass data peak lists for the Mascot searches were generated using Sequest in Qual Browser software (Bioworks Browser 3.2, Excalibur, Thermo) or with Proteome Discoverer.
5. Peptide modifications were included in the search as variables (e.g. oxidation on methionine, histidine, or tryptophan; ubiquitination on lysine and N-terminus amino acid; acetylation on N-terminus amino acid).
6. Mascot threshold cutoffs for acceptable identified peptides have MASCOT scores > 20, mass errors < 3 ppm, and expect values < 1.

## Supporting Information

**Supplementary Legends**

**Review Process File**
