# Supplementary material for: TRIM5α requires Ube2W to anchor Lys63-linked ubiquitin chains and restrict reverse transcription
Source: EMBO J. 2015 Jun 22;34(15):2078–95. doi: 10.15252/embj.201490361 (PMC4551353; doi:10.15252/embj.201490361)
Supplement: Supplementary file 1 [file embj0034-2078-sd1.pdf]

Figure S1

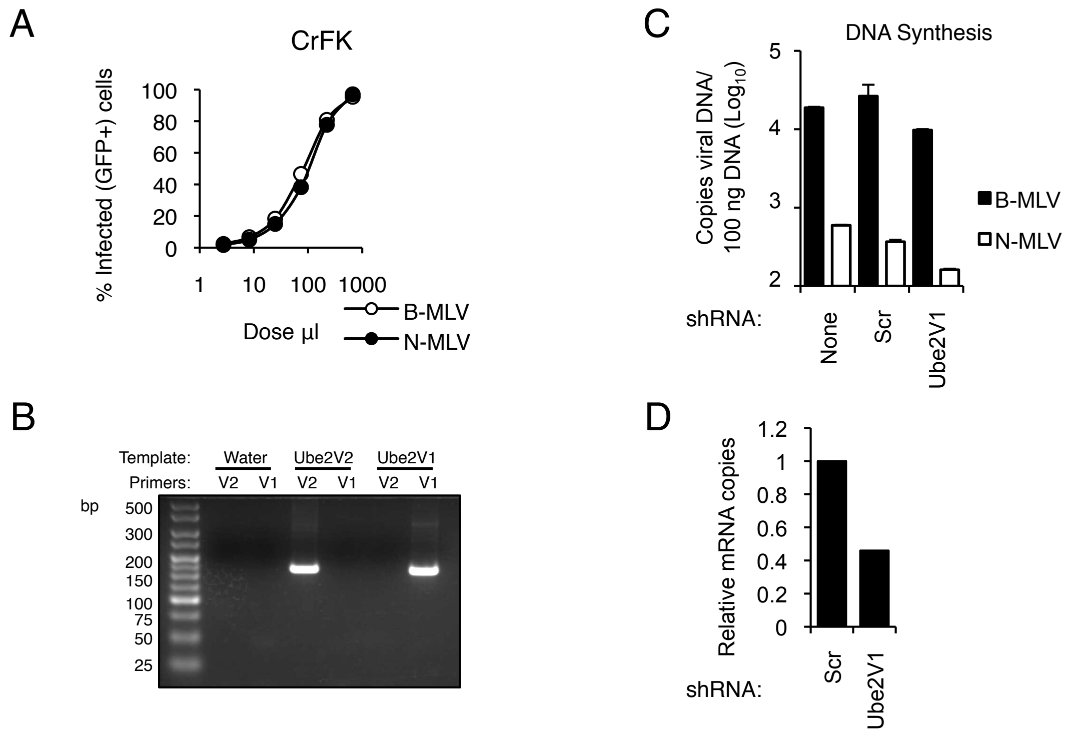
A
CrFK
% Infected (GFP+) cells
100
80
60
40
20
0
1
10
100
1000
Dose µl
B-MLV
N-MLV
B
Template:
Water
Ube2V2
Ube2V1
Primers:
V2
V1
V2
V1
V2
V1
bp
500
300
200
150
100
75
50
25
C
DNA Synthesis
Copies viral DNA/
100 ng DNA (Log10)
5
4
3
2
B-MLV
N-MLV
shRNA:
None
Scr
Ube2V1
D
Relative mRNA copies
1.2
1
0.8
0.6
0.4
0.2
0
shRNA:
Scr
Ube2V1

Figure S2A

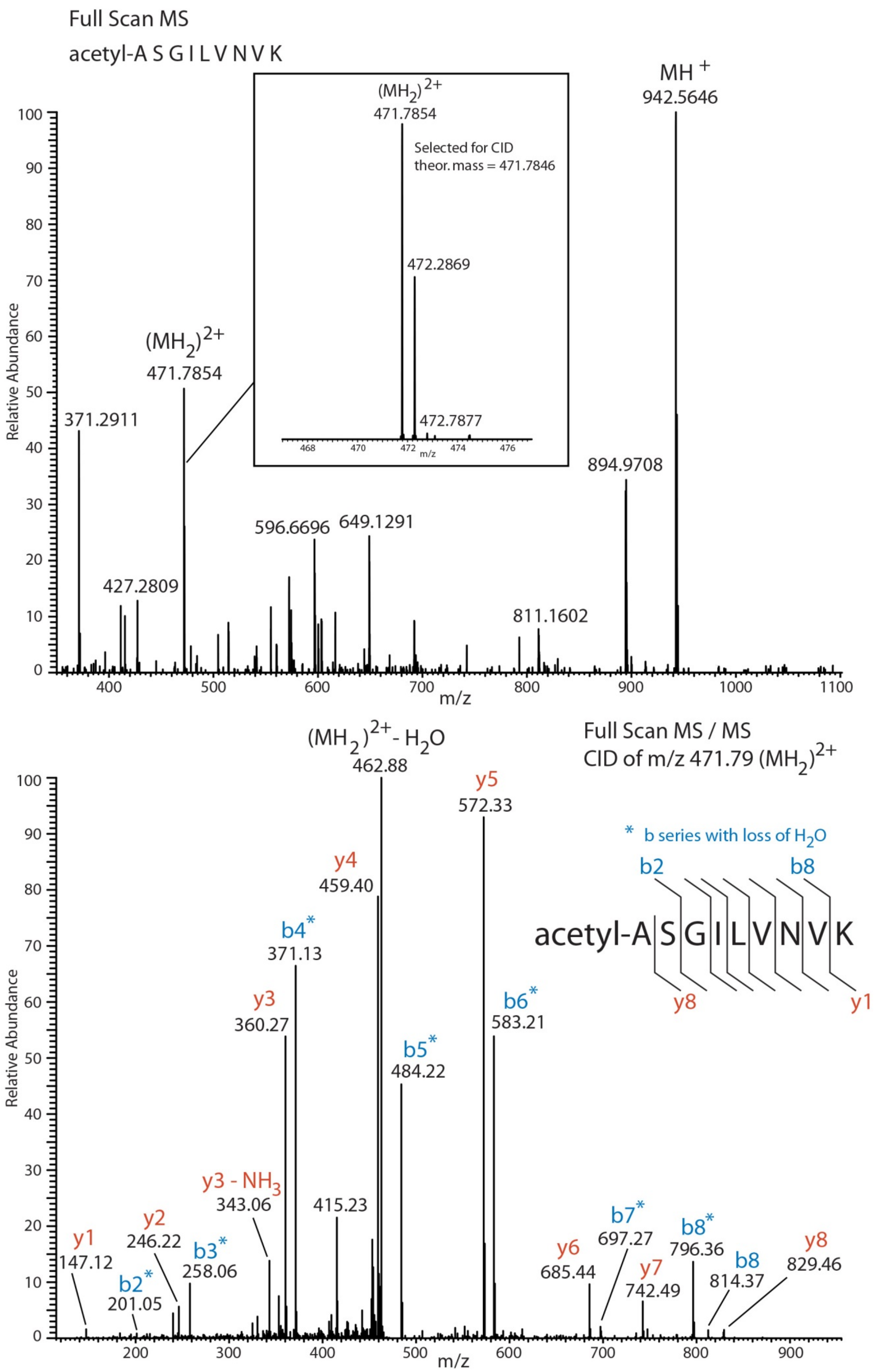
Full Scan MS
acetyl-A S G I L V N V K
MH +
942.5646
(MH2)2+
471.7854
Selected for CID
theor. mass = 471.7846
472.2869
472.7877
371.2911
427.2809
596.6696
649.1291
811.1602
894.9708
Relative Abundance
m/z
(MH2)2+- H2O
462.88
Full Scan MS / MS
CID of m/z 471.79 (MH2)2+
* b series with loss of H2O
acetyl-A S G I L V N V K
b2
b8
y8
y1
y5
572.33
y4
459.40
b4*
371.13
y3
360.27
b6*
583.21
b5*
484.22
y3 - NH3
343.06
415.23
y1
147.12
y2
246.22
b2*
201.05
b3*
258.06
y6
685.44
b7*
697.27
y7
742.49
b8*
796.36
b8
814.37
y8
829.46

Figure S2B

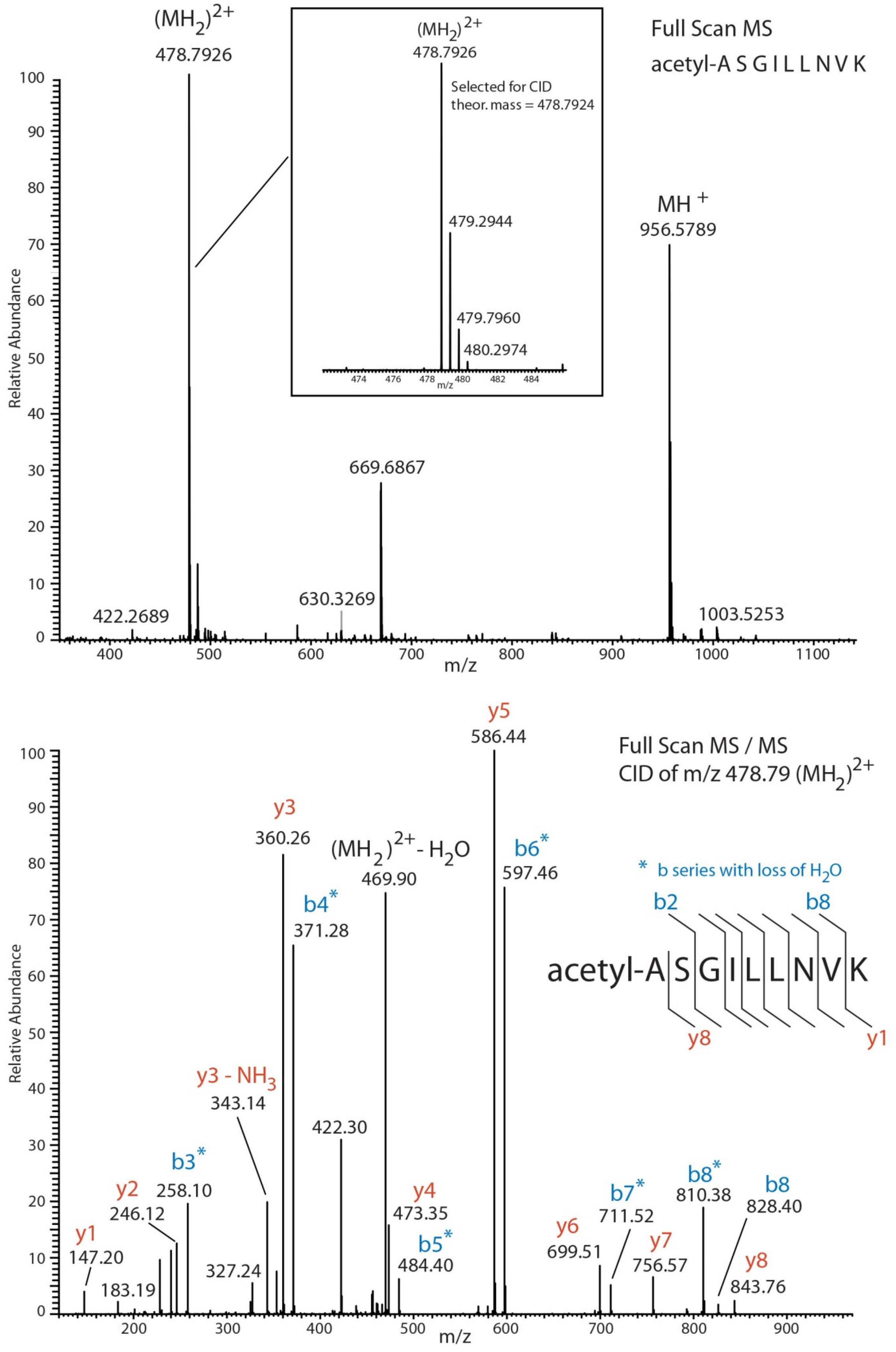

Full Scan MS
acetyl-A S G I L L N V K
(MH2)2+
478.7926
Selected for CID
theor. mass = 478.7924
479.2944
479.7960
480.2974
MH +
956.5789
669.6867
630.3269
422.2689
1003.5253
Relative Abundance
m/z
Full Scan MS / MS
CID of m/z 478.79 (MH2)2+
* b series with loss of H2O
acetyl-A S G I L L N V K
y5 586.44
y3 360.26
(MH2)2+- H2O 469.90
b6* 597.46
b4* 371.28
y3 - NH3 343.14
422.30
b3* 258.10
y2 246.12
y1 147.20
183.19
327.24
y4 473.35
b5* 484.40
y6 699.51
b7* 711.52
y7 756.57
b8* 810.38
b8 828.40
y8 843.76
b2
y8
y1

Figure S3

A

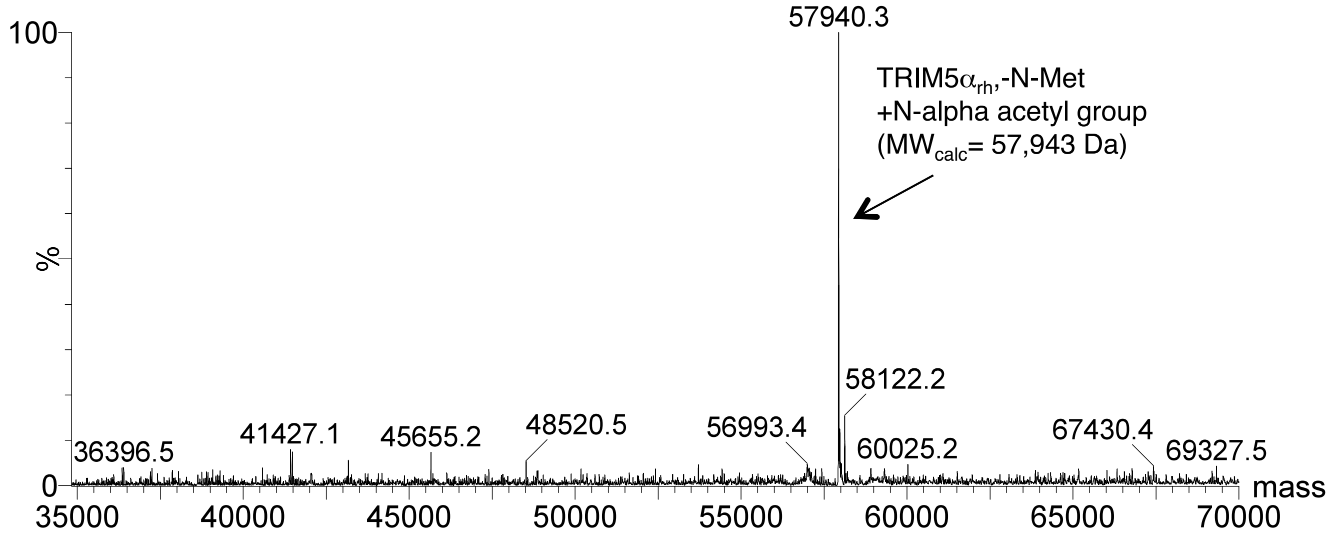

57940.3
TRIM5αrh,-N-Met
+N-alpha acetyl group
(MWcalc= 57,943 Da)
100
%
0
58122.2
36396.5
41427.1
45655.2
48520.5
56993.4
60025.2
67430.4
69327.5
mass
35000
40000
45000
50000
55000
60000
65000
70000

B

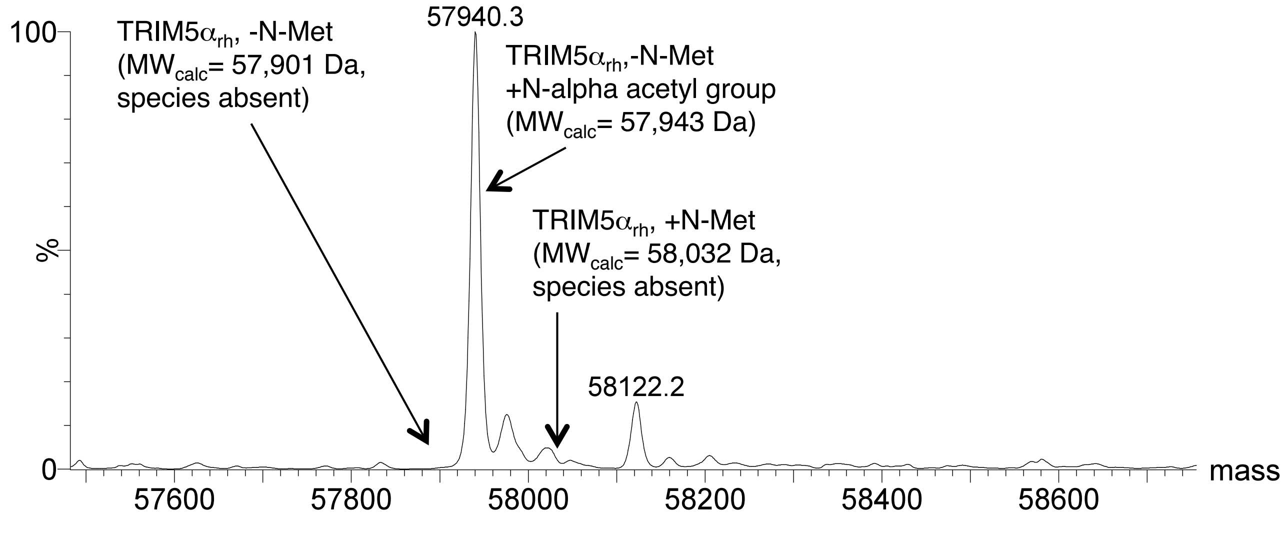

100
TRIM5αrh, -N-Met
(MWcalc= 57,901 Da,
species absent)
57940.3
TRIM5αrh,-N-Met
+N-alpha acetyl group
(MWcalc= 57,943 Da)
%
TRIM5αrh, +N-Met
(MWcalc= 58,032 Da,
species absent)
58122.2
0
mass
57600
57800
58000
58200
58400
58600

Figure S4

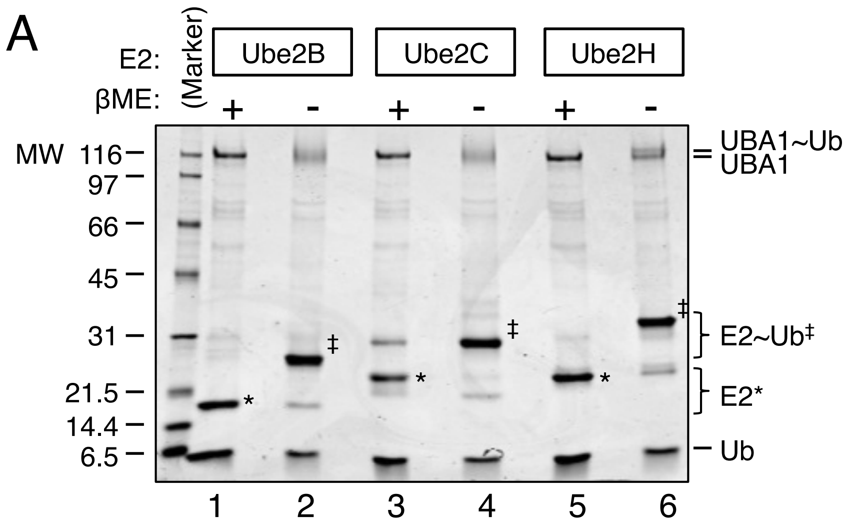

A
E2:
(Marker)
Ube2B
Ube2C
Ube2H
βME:
+ - + - + -
MW 116
97
66
45
31
21.5
14.4
6.5
UBA1~Ub
UBA1
E2~Ub‡
E2*
Ub
1 2 3 4 5 6

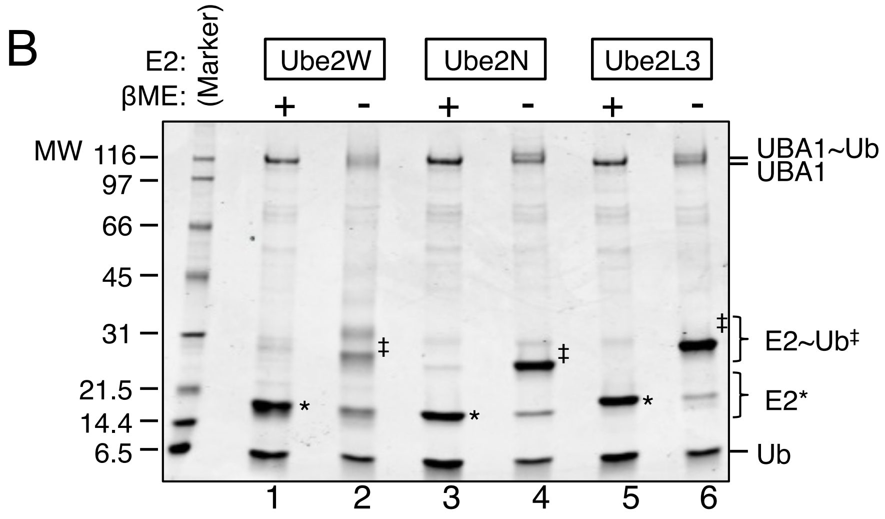

B
E2:
(Marker)
Ube2W
Ube2N
Ube2L3
βME:
+ - + - + -
MW 116
97
66
45
31
21.5
14.4
6.5
UBA1~Ub
UBA1
E2~Ub‡
E2*
Ub
1 2 3 4 5 6

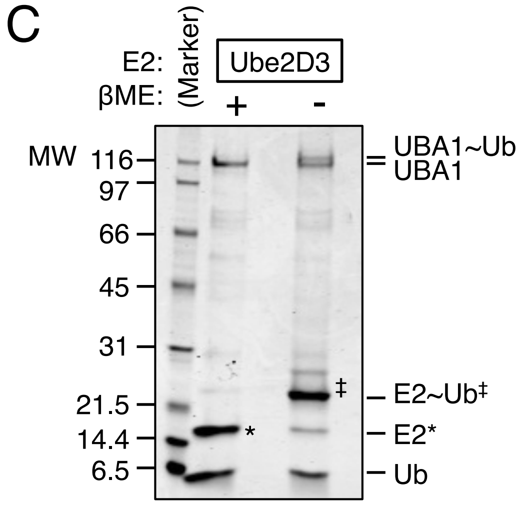

C
E2:
(Marker)
Ube2D3
βME:
+ -
MW 116
97
66
45
31
21.5
14.4
6.5
UBA1~Ub
UBA1
E2~Ub‡
E2*
Ub
1 2

Figure S5

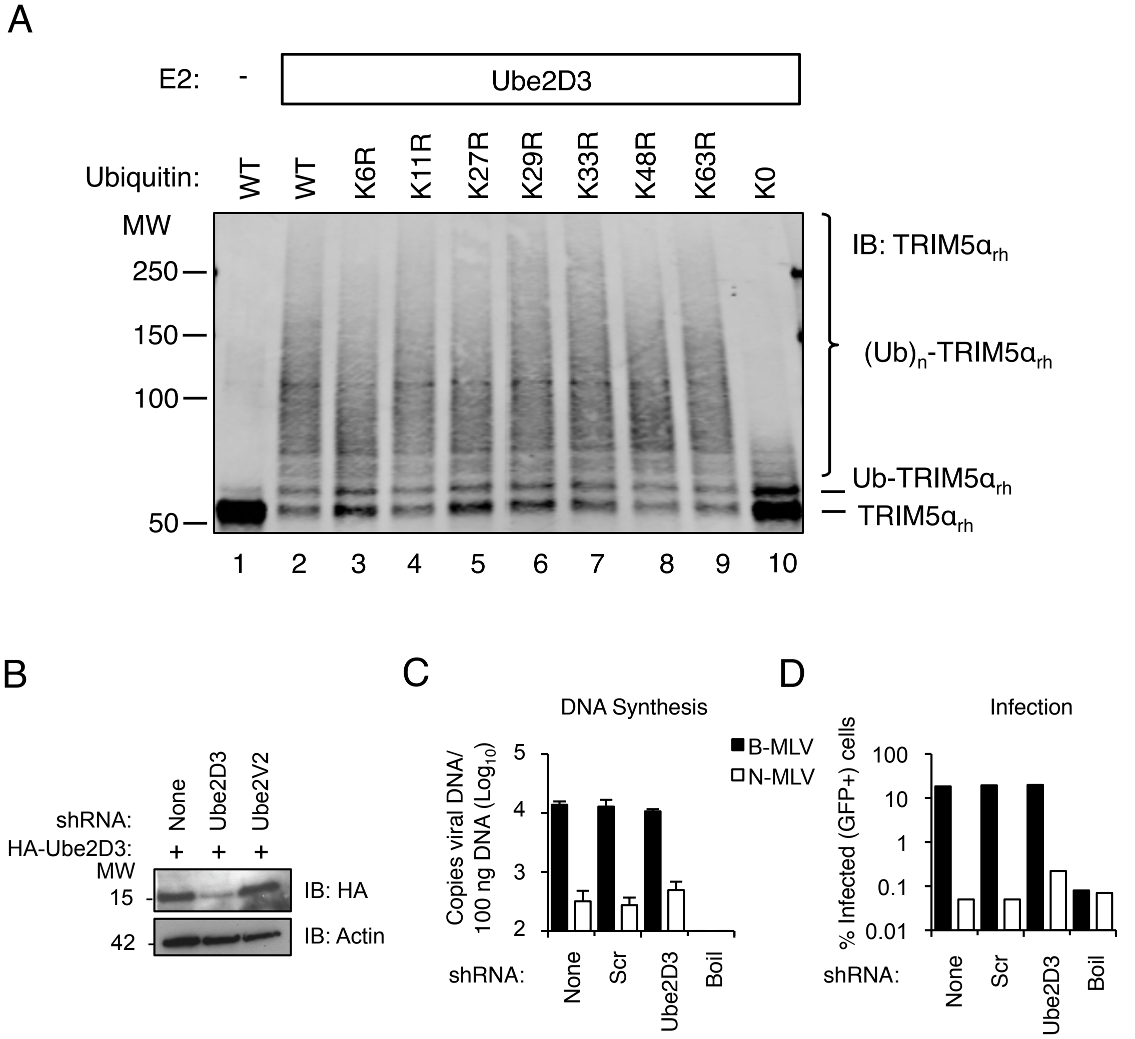
A
E2:
-
Ube2D3
Ubiquitin:
WT
WT
K6R
K11R
K27R
K29R
K33R
K48R
K63R
K0
MW
250
150
100
50
IB: TRIM5α rh
(Ub)n-TRIM5α rh
Ub-TRIM5α rh
TRIM5α rh
1 2 3 4 5 6 7 8 9 10
B
shRNA:
None
Ube2D3
Ube2V2
HA-Ube2D3:
+ + +
MW
15
42
IB: HA
IB: Actin
C
DNA Synthesis
Copies viral DNA/ 100 ng DNA (Log10)
5
4
3
2
B-MLV
N-MLV
shRNA:
None
Scr
Ube2D3
Boil
D
Infection
% Infected (GFP+) cells
100
10
1
0.1
0.01
shRNA:
None
Scr
Ube2D3
Boil

Figure S6

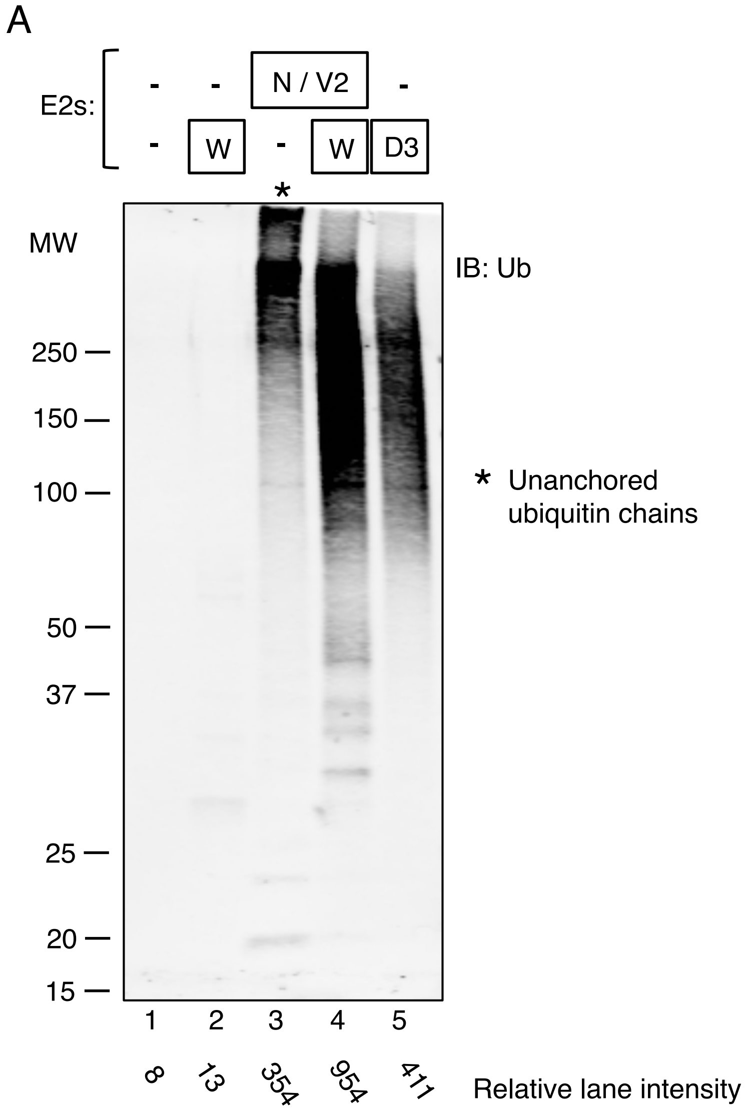
A
E2s:
-
-
N / V2
-
-
W
-
W
D3
*
MW
IB: Ub
250
150
100
50
37
25
20
15
* Unanchored ubiquitin chains
1
2
3
4
5
8
13
354
954
411
Relative lane intensity

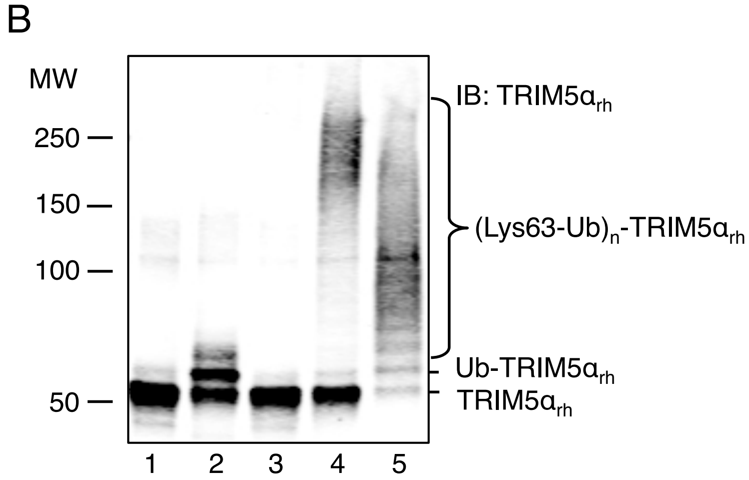
B
MW
IB: TRIM5α$_{rh}$
250
150
100
50
(Lys63-Ub)$_n$-TRIM5α$_{rh}$
Ub-TRIM5α$_{rh}$
TRIM5α$_{rh}$
1
2
3
4
5

Figure S7

A

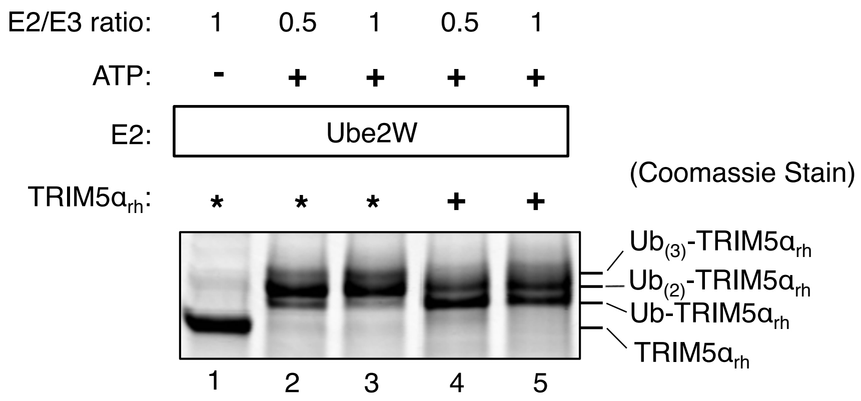

E2/E3 ratio: 1 0.5 1 0.5 1
ATP: - + + + +
E2: Ube2W
TRIM5αrh: * * * + +
(Coomassie Stain)
Ub(3)-TRIM5αrh
Ub(2)-TRIM5αrh
Ub-TRIM5αrh
TRIM5αrh
1 2 3 4 5

B

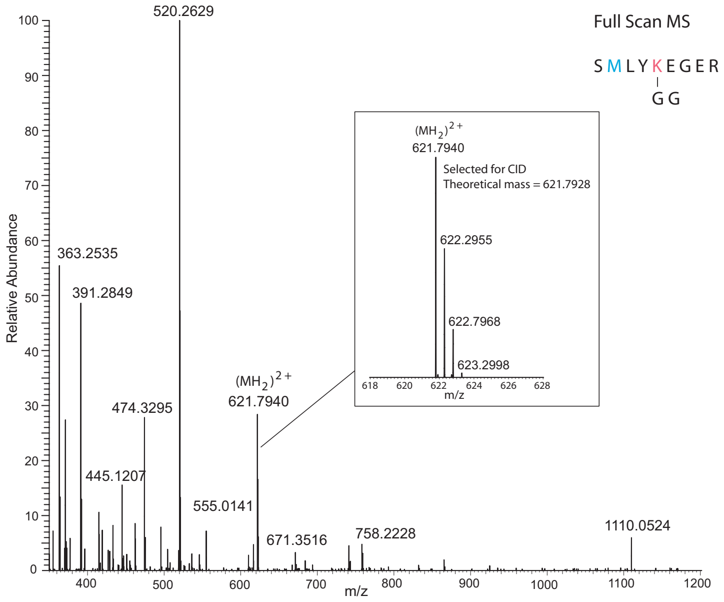

Full Scan MS
SMLYKEGER
GG
Relative Abundance
m/z
520.2629
363.2535
391.2849
474.3295
445.1207
555.0141
(MH2)2+
621.7940
671.3516
758.2228
1110.0524
Selected for CID
Theoretical mass = 621.7928
622.2955
622.7968
623.2998

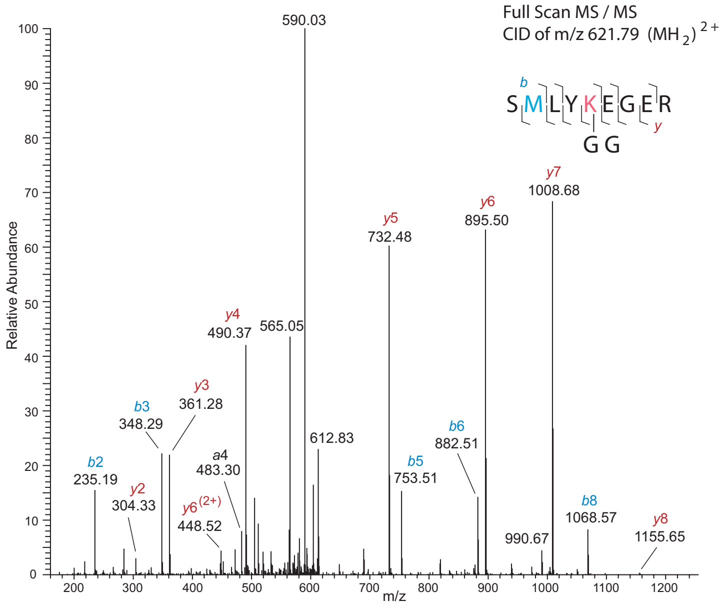

Full Scan MS / MS
CID of m/z 621.79 (MH2)2+
SMLYKEGER
GG
Relative Abundance
m/z
590.03
y7 1008.68
y6 895.50
y5 732.48
y4 490.37
565.05
y3 361.28
b3 348.29
612.83
b6 882.51
b5 753.51
a4 483.30
b2 235.19
y2 304.33
y6(2+) 448.52
b8 1068.57
y8 1155.65
990.67

Figure S8

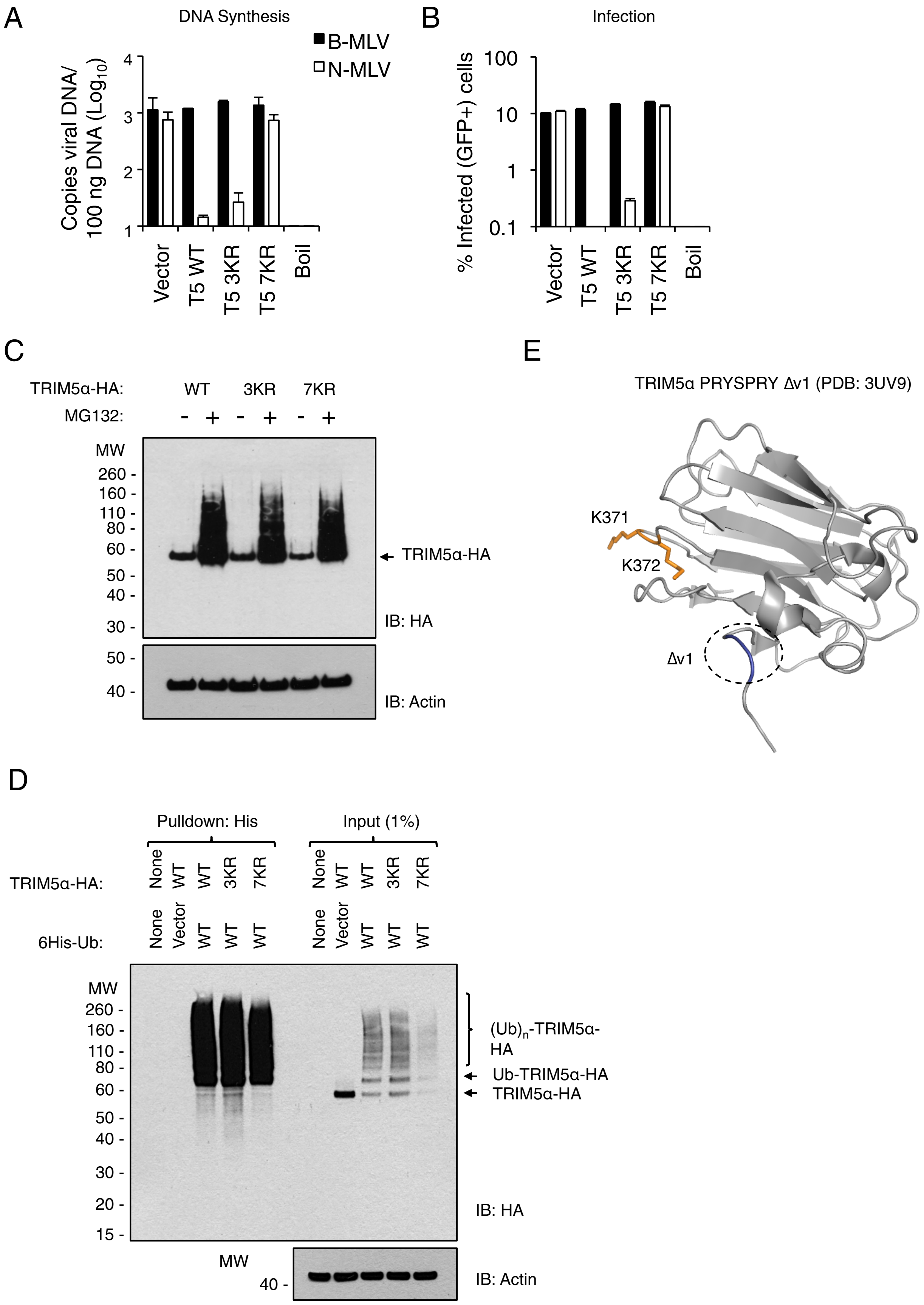

A
DNA Synthesis
B-MLV
N-MLV
Copies viral DNA/ 100 ng DNA (Log_{10})
4
3
2
1
Vector
T5 WT
T5 3KR
T5 7KR
Boil
B
Infection
% Infected (GFP+) cells
100
10
1
0.1
Vector
T5 WT
T5 3KR
T5 7KR
Boil
C
TRIM5α-HA:
WT
3KR
7KR
MG132:
- + - + - +
MW
260 -
160 -
110 -
80 -
60 -
50 -
40 -
30 -
TRIM5α-HA
IB: HA
50 -
40 -
IB: Actin
E
TRIM5α PRYSPRY Δv1 (PDB: 3UV9)
K371
K372
Δv1
D
Pulldown: His
Input (1%)
TRIM5α-HA:
None
WT
WT
3KR
7KR
None
WT
WT
3KR
7KR
6His-Ub:
None
Vector
WT
WT
WT
None
Vector
WT
WT
WT
MW
260 -
160 -
110 -
80 -
60 -
50 -
40 -
30 -
20 -
15 -
$(Ub)_n$-TRIM5α-HA
Ub-TRIM5α-HA
TRIM5α-HA
IB: HA
MW
40 -
IB: Actin

Table S1. Expression Plasmids, Antibodies, siRNAs, Oligonucleotides, Real-Time Probes Used in This Study

1A. Bacterial Expression Vectors

Plasmid Name	Source[1]	Internal ID	Cloning Site	Epitope Tags
pET11HisPP-Ube2B	NP_003328	WISP11-266	*NheI-XhoI*	His-PP
pET11HisPP-Ube2C	NP_008950	WISP11-267	*NheI-XhoI*	His-PP
pET11HisPP-Ube2D3	NP_003331	WISP14-34	*NheI-XhoI*	His-PP
pET11HisPP-Ube2H	NP_003335	WISP11-270	*NheI-XhoI*	His-PP
pET11HisPP-Ube2L3	NP_003338	WISP14-35	*NheI-XhoI*	His-PP
pET11HisPP-Ube2N	NP_003339	WISP11-269	*NheI-XhoI*	His-PP
pET11HisPP-Ube2V1	NP_068823	WISP14-36	*NheI-XhoI*	His-PP
pET11HisPP-Ube2V2	NP_003341	WISP11-272	*NheI-XhoI*	His-PP
pET11HisPP-Ube2W	Q96B02-2	WISP14-37	*NheI-XhoI*	His-PP
pET15b-Ubiquitin	Rachel Klevit	WISP11-276	*BamHI-KpnI*	none
pET15b-Ubiquitin K6R	WISP11-276	WISP11-277	*BamHI-KpnI*	none
pET15b-Ubiquitin K11R	WISP11-276	WISP14-38	*BamHI-KpnI*	none
pET15b-Ubiquitin K27R	WISP11-276	WISP14-39	*BamHI-KpnI*	none
pET15b-Ubiquitin K29R	WISP11-276	WISP14-40	*BamHI-KpnI*	none
pET15b-Ubiquitin K33R	WISP11-276	WISP14-41	*BamHI-KpnI*	none
pET15b-Ubiquitin K48R	WISP11-276	WISP11-278	*BamHI-KpnI*	none
pET15b-Ubiquitin K63R	WISP11-276	WISP11-279	*BamHI-KpnI*	none
pET15b-Ubiquitin K0	WISP11-276	WISP14-42	*BamHI-KpnI*	none
pET21d-UBA1	Cynthia Wolberger	WISP14-43		His

1B. Insect Expression Vectors

Plasmid Name	Source[1]	Internal ID	Cloning Site	Epitope Tags
pFastBac1 OSF-PP-TRIM5a (rhesus)	DQ842021	WISP13-56	*SLIC*	OSF-PP

1C. Mammalian Expression Vectors

Plasmid Name	Source[1]	Internal ID	Cloning Site	Epitope Tags
pHR-SIN-6xHis-UbGFP-WT	Paul Lehner		*BamHI-NotI*	His
pHR-SIN-6xHis-UbGFP-K48R	Paul Lehner		*BamHI-NotI*	His

pHR-SIN-6xHis-UbGFP-K63R	Paul Lehner		*BamHI-NotI*	His
EXN	Paul Bieniasz		*EcoRI-NotI*	HA
pcDNA3.1(+)	Invitrogen		*EcoRI-NotI*	
pcDNA4-His	Invitrogen		*EcoRI-NotI*	His
pSIREN-RetroQ	Clontech	SRQ	*BamHI-EcoRI*	None
pMT123	(Treier et al, 1994)	HA-Ub		
pMT107	(Treier et al, 1994)	His-Ub		

1D. Virus Production Vectors

Plasmid Name	Source[1]	Internal ID	Cloning Site
pCMVi	Francoise Loic Cosset		
pCIG3-N	(Bock et al, 2000)		
pCIG3-B	(Bock et al, 2000)		
pCNCG	Yasu Takeuchi		
p8.91Ex	Yasu Ikeda		
pMD2.G	(Naldini et al, 1996)		

1E. Antibodies

Antigen	Species	Blocking	Dilution	*Source*
TRIM5	Rhesus	2% milk	1/1000	*Lampire Biological Laboratories, NIH Reagent Program (5D5-1-1)*
HA-tag	Rat	1% milk	1/1000	*Roche, 3F10*
Ube2N	Rabbit	1% milk	1/1000	*Millipore, AB10025*
β-Actin	Mouse	None	1/40000	*Abcam, AC-15*
His-tag	Mouse	3% milk	1/1000	*Millipore, H8*
Ubiquitin	Mouse	2% milk	1/2000	*Santa Cruz Biotechnology, P4D1*
Ub Lys63	Rabbit	5% BSA	1/2000	*Millipore, Apu3*

1F. shRNA 19mers

Protein	First nucleotide	Sense sequence	*Reference*
Ube2W	920 (3'UTR)	GCATGATAGGGCCTATGAA	*This study*
Ube2V1	1409 (3'UTR)	CCCTGGTTTCTTTAAGTCTTAA	*(Pertel et al, 2011)*
Ube2V2	324 (ORF)	GAGCATACCAGTGTTAGCA	*This study*
Ube2D3	237 (ORF)	CAGTAATGGCAGCATTTGT	*(Saville et al, 2004)*
Ube2N	788 (ORF)	GAGCATGGACTAGGCTATA	*(Duncan et al, 2006)*
Scrambled		GTTATAGGCTCGCAAAAGG	*This study*

1G. Cloning Oligonucleotides

Target	Sense sequence	Anti-sense sequence
TRIM5α_{hu}	ATGCCAATTGATGGCTTCTGGAATCCTGGTTAATGTAAAGG	ATCGGCGGCCGCTCAAGAGCTTGGTGAGCACAGAG
Ube2V2	CAGTGAATTCCACCATGGCGGTCTCCACAGGAGTTAAAG	CGATGCGGCCGCTTAATTGTTGTATGTTTGTCCTTCTGGTGGCTG
Ube2V1	ATGCGGATCCATGCCAGGAGAGGTTCAAGCGTC	ATGCGCGGCCGCTTAATTGCTGTAACACTGTCCTTCG
Ube2W	ATGCGAATTCATGGCGTCAATGCAGACCACAG	ATGCGCGGCCGCTCAACAAGTATCATCATG

1H. Real-Time Oligonucleotides and Probe

Target	Sense sequence	Anti-sense sequence
GFP	CAACAGCCACAACGTCTATATCAT	ATGTTGTGGCGGATCTTGAAG
GFP probe	5'[6FAM]CCGACAAGCAGAAGAACGGCATCAA[TAM]3'	
Ube2V2	CCACCAAGGACAAATTATG	TGCTAACACTGGTATGCTCC
Ube2V1	GGGCCTCCAAGAACAATTTATG	CTGATATGGCTCTTGGGTCC
GAPDH	GGCTGAGAACGGGAAGCTT	AGGGATCTCGCTCCTGGAA
Ube2W	CTCTCCTCAGGTCATGTTTAC	GAATTATCCGGTGGTCGTC

[1] Source refers to vendors for commercial vectors, papers describing these constructs or the Uniprot accession numbers used for cloning.

Abbreviations
SLIC, Sequence and ligation independent cloning
OSF, One STrEP and FLAG tag
PP, PreScission Protease Cleavage site
