# Supplementary material for: TRIM5α requires Ube2W to anchor Lys63-linked ubiquitin chains and restrict reverse transcription
Source: EMBO J. 2015 Jun 22;34(15):2078–95. doi: 10.15252/embj.201490361 (PMC4551353; doi:10.15252/embj.201490361)
Supplement: Supplementary file 2 [file embj0034-2078-sd2.docx]

**Supplementary Figure Legends**

**Figure S1. Controls for vector titers and qPCR primer specificities. Related to Figure 1.**

A N- and B-MLV GFP vectors are equally infectious in feline CrFK cells ([McEwan et al, 2009](#_ENREF_27)). Solutions containing equal concentrations of N- or B-MLV-GFP vectors were titrated on CrFK cells and the percent infection (flow cytometry for GFP) was quantified 48 hr post infection (p.i.).

B PCR primers used to quantify mRNA levels of the enzymes Ube2V1 and Ube2V2 in Fig. 1A are not cross-reactive. PCR reactions containing the designated enzyme primer pair (Ube2V1 = V1, Ube2V2 = V2), an E2 cDNA template or water, were performed and the products were separated on an agarose gel. The data demonstrate that Ube2V1 primers only recognize Ube2V1 cDNA and Ube2V2 primers only recognize Ube2V2 cDNA.

C TE671 cells that stably expressed Ube2V1-specific shRNA, or a scrambled control shRNA (Scr), were transduced with N- or B-MLV-GFP vectors. Copies of viral DNA at 6 hr post infection (p.i.) (TaqMan GFP qPCR) were determined in parallel samples. Boiled virus served as a negative control. Mean ± SEM of duplicates.

D mRNA levels of Ube2V1 were assessed by RT-qPCR in TE671 cells that stably expressed Ube2V1-specific shRNA, or Scr. Values were calculated relative to GAPDH mRNA and expressed relative to levels in cells expressing Scr control.

**Figure S2.**  **LC-MS-MS mass spectrometry of TRIM5α proteins.**

Human and Rhesus TRIM5α proteins were exogenously expressed in Human 293T cells with a C-terminal FOS tag. TRIM5α-FOS proteins were purified by Streptactin affinity and the nature of the N-terminus of the TRIM5α proteins was determined by LC-MS-MS.

A (upper panel) MS spectra of the only identified N-terminal peptide from Human TRIM5α-FOS and the MS-MS spectra (lower panel) of the fragmentation pattern showing a positive identification of a de-methionylated and *α*N-acetyled TRIM5α_hu_ N-terminal peptide.

B Similar data is presented for TRIM5α_rh_ upper and lower panels. N-terminal peptides with an intact methionine or peptides that were de-methionylated and not acetylated were not detected.

**Figure S3.** **Electrospray mass spectrometry of purified recombinant TRIM5α_rh_ protein.**

A After removal of the C-terminal FOS tag by precision protease, a non-native six residue “LEVLFQ” sequence remains at the C-terminus, with the N-terminal Met and no post-translational modifications, resulting in a protein with an expected mass of 58,032 Daltons. Removal of the N-terminal Met residue and addition of an acetyl group results in a TRIM5α_rh_ protein with an expected mass of 57,943 Daltons, which is consistent with the observed peak of 57940.3 Daltons because the accuracy of the intact measurement is typically better than 3 Da for a protein with a mass of 50K Da.

B An expanded region of the mass spectrum focusing on the peak for TRIM5α_rh_. Note also the absence of species corresponding to unacetylated, demethionylated TRIM5α or methionylated TRIM5α.

**Figure S4. Each E2 is actively charged with ubiquitin by UBA1**

A-C Each purified E2 was incubated with UBA1 (E1), Ub, and ATP for 15 minutes at 37°C. After the incubation, samples were divided and mixed with 2x SDS sample buffer with or without reducing agent (β-mercaptoethanol, βME) and reactions products separated by SDS-PAGE. UBA1 charges the E2 with Ub via a thiol-ester bond (E2~Ub^‡^) between the C-terminal Gly residue of Ub and the active site cysteine of the E2 that is sensitive to reducing agents (E2*).

**Figure S5.** **TRIM5α_hu_ restriction of N-MLV DNA synthesis is not affected by Ube2D3 depletion. Related to Figure 5.**

A Ube2D3 forms multiple different types of polyUb-linked chains on TRIM5α_rh_. TRIM5α_rh_ (0.4 μM) was incubated with Ube2D3 (0.4 μM), UBA1 (0.2 μM), ATP (5 mM), and either wild-type Ub or Ub proteins with the designated Lys to Arg mutations (10 μM). K0 Ub has all 7 lysine residues mutated to arginine. TRIM5α_rh_ products were analyzed by immunoblot (IB) detection (TRIM5α_rh_). Representative of two experiments.

B The efficacy and specificity of Ube2D3-depletion was confirmed by IB of TE671 cells that were transfected with expression constructs for HA-tagged Ube2D3 and that stably expressed shRNAs against Ube2D3 or Ube2V2, or no shRNA (denoted None). IBs show expression of HA-Ube2D3 (HA), and loading control β-actin.

C and D Wild type TE671 cells or TE671 cells stably expressing Ube2D3-specific shRNA or a scrambled shRNA (Scr), were transduced with N- or B-MLV-GFP vectors (MOI ~ 0.2). Parallel samples were assayed for copies of viral DNA at 6 hr p.i. (TaqMan GFP qPCR) (C) and percent infection at 48 hr p.i. (flow cytometry for GFP) (D). Boiled virus served as a negative control. Mean ± SEM of triplicates.

**Figure S6.** **Ube2W alters the ubiquitination behavior of Ube2N/Ube2V2 with TRIM5α. Related to Figure 6.**

A and B Comparative analysis of polyUb products of Ube2N/Ube2V2 generated with TRIM5α_rh_ in the presence or absence of Ube2W. The assays were conducted under the same conditions as the reactions in Fig. 6, and the reactions products were analyzed by immunoblot (this figure) detecting Ub (A) or TRIM5α_rh_ (B), and LC-MS-MS (Table 1).

**Figure S7.** **Mapping of sites of TRIM5α_rh_ ubiquitination.**

A Preparative-scale Ub reactions with TRIM5α_rh_ and Ube2W. TRIM5α_rh_ (4 μM) was incubated with Ube2W (2 μM or 4 μM), Ub (10 μM), UBA1 (2 μM), and ATP (5 mM) at 37°C for 60 minutes. Reaction products were separated by SDS-PAGE and stained with Coomassie Brilliant Blue. Gel bands representing ubiquitinated forms of TRIM5αrh were excised, trypsinized, and analyzed by LC-MS-MS for sites of Ub attachment to TRIM5α_rh_. TRIM5α_rh_ substrates in lanes 1-3 have an artificial, free N-terminus (designated *TRIM5α_rh_) whereas TRIM5α_rh_ substrate in lanes 4 and 5 have the native, acetylated N-terminus (designated +, see text for details).

B MS (upper) and MS-MS (lower) spectra showing the definitive identification of the TRIM5α_rh_ ubiquitination site at Lys50. The trypsin-digested peptide, from lane 5 in (A), corresponds to the peptide: _46_SM_ox_LYK(GG)EGER_54_ where M_ox_ represents an oxidized Met47 residue and (GG) represents the Gly-Gly dipeptide that remains linked via an isopeptide bond to Lys50 following trypsin digestion. Ubiquitinated Lys45 peptide was also identified in the analyses of the reaction mixtures excised from lanes 4 and 5, as was a peptide corresponding to a K48-linked Ub dipeptide (not shown). Ub modification of Lys284 was also detected in other repetitions of this experiment. As expected, a peptide corresponding to the demethionylated, acetylated N-terminus was detected, and no other N-terminal peptides were detected. Analyses of the reaction mixtures in lanes 2 and 3 indicated the presence of Ub linkages at the (free) N-terminus of TRIM5α_rh_ and at internal Lys residues 45, 50, 85, 218 and 372.

**Figure S8.** **Mutation of TRIM5α_hu_ lysine residues does not impair cellular ubiquitnation**

A and B CrFK cells stably expressing empty vector EXN or C-terminal HA-tagged TRIM5α_hu_ WT or bearing lysine-to-arginine substitutions 3KR: K44R, K45R, K50R or 7KR: K44R, K45R, K50R, K84R, K282R, K367R, K368R, were transduced with N- or B-MLV-GFP vectors. Copies of viral DNA at 6 hr post infection (p.i.) (TaqMan GFP qPCR) (A) and percent infection at 48 hr p.i. (flow cytometry for GFP) (B), determined in parallel samples. Boiled virus served as a negative control. Mean ± SEM n=2.

C CrFK cells expressing WT or mutant TRIM5α_hu_-HA variants from (A) were treated with the proteasome inhibitor MG132, lysed at the indicated time intervals, and analyzed by IB detection of the HA-tag (HA), or β-actin (loading control).

D 293T cells were transfected with the designated combinations of expression constructs for 6xHis-Ub and WT or mutant TRIM5α_hu_-HA. After 48 hr, 6xHis-Ub was pulled down (PD) with a Ni^2+^ affinity matrix, followed by analysis of the input and affinity purified samples by IB with antibodies detecting the HA-tag (HA) or β-actin (loading control). All blots are representative of at least two independent experiments. In this experiment cells were not treated with MG132.

E Crystal structure of the PRYSPRY domain from TRIM5α_rh_, PDB code 3UV9. The first variable loop (v1) was deleted to aid crystallization ([Biris et al, 2012](#_ENREF_6)). Exposed side chains of lysine residues 371 and 372 (corresponding to TRIM5α_hu_ lysine residues 367 and 368) are highlighted. The dotted circle identifies the alanine-glycine motif substituted for v1.
