# Supplementary material for: TRIM5α requires Ube2W to anchor Lys63-linked ubiquitin chains and restrict reverse transcription
Source: EMBO J. 2015 Jun 22;34(15):2078–95. doi: 10.15252/embj.201490361 (PMC4551353; doi:10.15252/embj.201490361)
Supplement: Supplementary file 3 [file embj0034-2078-sd3.pdf]

THE EMBO JOURNAL

Manuscript EMBO-2014-90361

TRIM5 requires Ube2W to anchor Lys63-linked ubiquitin chains and restrict reverse transcription

Adam J Fletcher, Devin E Christensen, Chad Nelson, Choon Ping Tan, Torsten Schaller, Paul J Lehner, Wesley I. Sundquist and Greg J Towers

Corresponding author: Greg Towers, MRC Centre for Medical Molecular Virology

Review timeline:

Submission date:	21 October 2014
Editorial Decision:	25 November 2014
Revision received:	23 April 2015
Editorial Decision:	12 May 2015
Revision received:	18 May 2015
Accepted:	20 May 2015

Editor: Karin Dumstrei

Transaction Report:

(Note: With the exception of the correction of typographical or spelling errors that could be a source of ambiguity, letters and reports are not edited. The original formatting of letters and referee reports may not be reflected in this compilation.)

1st Editorial Decision 25 November 2014

Thank you for submitting your manuscript to the EMBO Journal. Your study has now been seen by three experts and their comments are provided below.

As you can see, the referees find the analysis interesting and raise very similar points. The one major issue to resolve is to carry out mutational analysis of TRIM5α Lys45 and Lys50 residues and to test the ubiquitination status and antiretroviral activity of this mutant form of TRIM5α. There are some other issues raised as well, but they shouldn't be to difficult to sort out.

Should you be able to show that autoubiquitination of TRIM5α is important for its antiviral activity and address the other raised points then I would like to invite you to submit a revised version to us. I should point out that it is our policy to allow for one major round of revision only and that it is therefore important to address the raised concerns at this stage.

When preparing your letter of response to the referees' comments, please bear in mind that this will form part of the Review Process File, and will therefore be available online to the community. For more details on our Transparent Editorial Process, please visit our website: http://emboj.embopress.org/about#Transparent_Process

We generally allow three months as standard revision time. As a matter of policy,

competing manuscripts published during this period will not negatively impact on our assessment of the conceptual advance presented by your study. However, we request that you contact the editor as soon as possible upon publication of any related work, to discuss how to proceed. Should you foresee a problem in meeting this three-month deadline, please let us know in advance and we may be able to grant an extension.

Thank you for the opportunity to consider your work for publication. Should you have any further questions please don't hesitate to contact me.

REFEREE REPORTS

Referee #1:

TRIM5α, a RING-type ubiquitin E3 ligase, is of great interest because of its role in innate immunity and its ability to inhibit viral pathogens. How the TRIM5α ubiquitin ligase activity promotes those functions is an important unanswered question, as is if and how the proteasome system is involved. In this study, Fletcher et al. have made an important contribution by using a clever screen to identify the E2 enzymes that work with TRIM5α to restrict retroviral reverse transcription. The authors further showed that autoubiquitination by TRIM5α uses Ube2W to attached a single ubiquitin to an internal Lys; this monoubiquitination step is followed by K63-linked polyubiquitination by Ube2N-Ube2V2. Previously, Ube2W was thought to ubiquitinate exclusively α-amino groups at protein N-termini. These findings are important to better understand TRIM5α mechanism and function. However, the study falls a bit short in that the authors failed to show whether TRIM5α's autoubiquitination has functional significance for viral restriction. Also, although data were reported that implicate proteasome activity in the regulation of TRIM5α and its ubiquitination, but how those results might tie in to the rest of the story is unclear. Specific issues are detailed below.

1. Data from the screen of the 39 different E2 or UEV siRNAs (discussed on p. 5, top paragraph) are central to this paper and need to be included.
2. That Ube2V2 but not Ube2V1 functions with TRIM5α is interesting but also is a bit surprising. The validation done to confirm the role for Ube2V2 in viral restriction (p. 5, bottom) should also be done for Ube2V1. This is particularly important in light of the apparent equivalence of Ube2V1 and Ube2V2 in supporting TRIM5α autoubiquitination in vitro (Fig. 6A).
3. (Fig. 2; p. 6, lines 14-15 & 21-24) The authors interpret MG132-promoted accumulation of TRIM5α species as indicating proteasome-dependent turnover of TRIM5α, but that was never established. In fact, in Fig. 2A the total amounts of HA-TRIM5α species at 0 and 10 h of MG132 treatment appear to be similar (note the higher signal for the actin loading control at 10 h relative to 0 h). Similarly, the input samples in Fig. 2C show similar amounts of His-TRIM5α with and without MG132. The evidence does not support proteasome-dependent turnover; rather, the proteasome inhibition by MG132 could be having an indirect effect that causes the observed conjugate accumulation.
4. (Fig. 4A; p. 7, lines 21-23) The conclusion from this experiment, that Ub K63R expression blocked TRIM5α polyubiquitination, is unwarranted because the K63R ubiquitin was expressed at a much lower level than either WT or K48R. Also, why was non-ubiquitinated TRIM5α detected in the His-pulldown samples? Why was the 'non-specific' band intensity increased in lanes 2 & 3? Overall, the data in this figure appear unreliable.
5. (Fig. 5; p. 9, lines 7-10) The lack of apparent ligase activities with several E2 enzymes is inconclusive without accompanying evidence that the recombinant E2 enzymes used were, in fact, active.
6. The identification of the TRIM5α lysines (i.e., K45 & K50) targeted for

autoubiquitination provides an obvious opportunity to test the relevance of those sites for TRIM5α ubiquitination in cells and for their role in viral restriction. Those experiments are well within the scope of this study and should be done.
Some minor issues:
7. In Fig. 4E, "RLU" should be defined.
8. There are typos in many of the concentration units in Fig. 5
9. The magnified panel D in Fig. 3 is unnecessary.
10. (p. 8, line 35) Contrary to the claim, the ESI mass spectrum does not show definitively that 'demethionylation' and N-acetylation were 100%.
11. (p. 9, lines 1-5) The Pickart & Raasi paper does not describe purification of most of the E2 enzymes used by Fletcher et al. The sentence here is ambiguous.

Referee #2:

In this manuscript Fletcher and colleagues performed a targeted siRNA screen and show that the E2 enzymes Ube2W and Ube2N/Ube2V2 are required to inhibit N-MLV reverse transcription by human TRIM5α. The authors further propose a two-step ubiquitination mechanism in which Ube2W-dependent TRIM5α mono-ubiquitination is required for subsequent K63-linked TRIM5α polyubiquitination by the E2 Ube2N/Ube2V2 and TRIM5α-mediated suppression of viral DNA synthesis.
These findings have a significant impact on the field since the molecular mechanism of TRIM5α restriction, and in particular the involvement of TRIM5α ubiquitination in retrovirus restriction still remain poorly defined and somewhat controversial. The experiments are convincingly performed and clearly presented. However the authors should address the following points to confirm some of their findings.
Specific comments:
1) Does the inhibition of DNA synthesis shown in Fig. 1C correlate with virusinduced degradation of TRIM5α?
2) In Fig 2 the authors convincingly show that ectopically expressed TRIM5α is ubiquitinated and rapidly turned over by the proteasome. Can the authors provide any endogenous data to support this finding? Is the HMW TRIM5α which accumulates upon MG132 treatment (Fig. 2A) also detected by a K63UB specific antibody?
3) By mass spectrometry analysis the authors identified Lys45 and Lys50 as the residues on TRIM5α that are ubiquitinated on a Ube2W-dependent fashion. The authors should address ubiquitination status and antiretroviral activity of a TRIM5α protein mutated at these sites.

Referee #3:

This manuscript studies TRIM5alpha mediated antiviral activity. Using an siRNA screen the authors identify the E2 enzymes UBE2W and UBE2N/UBE2V2 (ubc13/mms2) as important for the ability of TRIM5a to abrogate reverse transcription, but not for its effect on infectivity.
They then show that these E2s are required to modify TRIM5alpha itself with UBE2W priming and Ubc13/mms2 extending the k63 chain. That is interesting as the UBE2W enzyme is known to modify N-terminal residues, whereas TRIM5alpha is acetylated at its N-terminus, preventing this modification. They then nicely show biochemically that UBE2W can place two ubiquitins at residues 45 and 50 if it is acetylated, and a third, when it is not.

The experiments are well-executed and the manuscript is well-written. To identify correct E2/E3 combinations is still quite a feat, and the data in this manuscript are convincing that

the link between TRIM5a and these E2s is real. Moreover, the ability of Ube2w to do more than target N-termini is very intriguing and will be important for understanding how this E2 functions.

What is missing is a functional link between the auto-ubiquitination of the ligase and its role in antiviral activity. At the very least the two acceptor lysines in Trim5alpha should be mutated, to see if it does play a role.
If that role is present, it would of course be very interesting to see what the K63 chains would bind to, but this may beyond the scope of the paper. However, if there is no functional link, one wonders what the target might be.

Minor points:
Page 8, line 22: unpublished observations? This does not seem appropriate, please just include the data here?
It is probably helpful to mention that UBE2N/UBE2V2 are UBC13/MMS2

1st Revision - authors' response 23 April 2015

Point by point rebuttal
"TRIM5α requires Ube2W to anchor Lys63-linked ubiquitin chains and restrict reverse transcription" EMBOJ-2014-90361

Referee #1:

TRIM5α, a RING-type ubiquitin E3 ligase, is of great interest because of its role in innate immunity and its ability to inhibit viral pathogens. How the TRIM5α ubiquitin ligase activity promotes those functions is an important unanswered question, as is if and how the proteasome system is involved. In this study, Fletcher et al. have made an important contribution by using a clever screen to identify the E2 enzymes that work with TRIM5α to restrict retroviral reverse transcription. The authors further showed that autoubiquitination by TRIM5α uses Ube2W to attached a single ubiquitin to an internal Lys; this monoubiquitination step is followed by K63-linked polyubiquitination by Ube2N-Ube2V2. Previously, Ube2W was thought to ubiquitinate exclusively α-amino groups at protein N-termini. These findings are important to better understand TRIM5α mechanism and function. However, the study falls a bit short in that the authors failed to show whether TRIM5α's autoubiquitination has functional significance for viral restriction. Also, although data were reported that implicate proteasome activity in the regulation of TRIM5α and its ubiquitination, but how those results might tie in to the rest of the story is unclear. Specific issues are detailed below.

1. Data from the screen of the 38 different E2 or UEV siRNAs (discussed on p. 5, top paragraph) are central to this paper and need to be included.

We have now included the data from the original screen as Fig 1A.

2. That Ube2V2 but not Ube2V1 functions with TRIM5α is interesting but also is a bit surprising. The validation done to confirm the role for Ube2V2 in viral restriction (p. 5, bottom) should also be done for Ube2V1. This is particularly important in light of the apparent equivalence of Ube2V1 and Ube2V2 in supporting TRIM5α autoubiquitination in vitro (Fig. 6A).

© European Molecular Biology Organization

We have now depleted Ube2V1 and shown that this does not rescue DNA synthesis of TRIM5 restricted MLV-N. We include 2 new panels in Fig E1C-D which show depletion of Ube2V1 RNA but no rescue of restricted MLV-N DNA synthesis.
*We include the text "*Because Ube2N can function with either Ube2V2 or Ube2V1 (Uev1a) (Hofmann & Pickart, 1999), we confirmed that depletion of Ube2V1 did not rescue restricted N-MLV DNA synthesis in HeLa cells (Fig. E1C-D).

3. (Fig. 2; p. 6, lines 14-15 & 21-24) The authors interpret MG132-promoted accumulation of TRIM5α species as indicating proteasome-dependent turnover of TRIM5α, but that was never established. In fact, in Fig. 2A the total amounts of HA-TRIM5α species at 0 and 10 h of MG132 treatment appear to be similar (note the higher signal for the actin loading control at 10 h relative to 0 h). Similarly, the input samples in Fig. 2C show similar amounts of His-TRIM5α with and without MG132. The evidence does not support proteasome-dependent turnover; rather, the proteasome inhibition by MG132 could be having an indirect effect that causes the observed conjugate accumulation.

We have now measured the relative amounts of modified and unmodified TRIM5α species on the IB in Fig 2A. The result confirms the reviewer's point that there is no increase of the unmodified TRIM5α band. However, HMW ubiquitinated species of TRIM5 clearly appear and accumulate after MG132 addition. Ie it is the ubiquitinated products that are now not degraded and therefore appear. We have now clarified this point and added the quantification to Fig 2A. We have changed the text to "A 2 hr MG132 treatment led to accumulation of higher molecular weight (HMW) HA-TRIM5α_{hu} species (Fig. 2A, lane 2). The HMW products continued to increase with time, ultimately becoming the predominant TRIM5α species after 10 hr of MG132 treatment (Fig. 2A, lane 5). Measurement of the HA band densities corresponding to the unmodified and HMW forms of HA-TRIM5α_{hu} demonstrated that it is not the unmodified form that accumulated in these experiments (Fig. 2A, bottom), rather it is the HMW species that accumulate, consistent with post-translational modification, such as ubiquitination."

4. (Fig. 4A; p. 7, lines 21-23) The conclusion from this experiment, that Ub K63R expression blocked TRIM5α polyubiquitination, is unwarranted because the K63R ubiquitin was expressed at a much lower level than either WT or K48R. Also, why was non-ubiquitinated TRIM5α detected in the His-pulldown samples? Why was the 'non-specific' band intensity increased in lanes 2 & 3? Overall, the data in this figure appear unreliable.

We agree that this experiment was less compelling than it should be. We have now repeated this experiment and include a new Fig 4A. In this case we have achieved better K63R ubiquitin expression as revealed by the IB panel detecting the Ub-HIS tag. We believe we can now show that despite equivalent K63R Ub expression we do not detect significant amounts of ubiquitinated TRIM5 when detecting the TRIM5 HA tag. Control lanes for wt and K48R Ub expression also demonstrate effective purification of ubiquitinated TRIM5 in these cases. We have also lost the non-specific bands on the HA IB.

5. (Fig. 5; p. 9, lines 7-10) The lack of apparent ligase activities with several E2 enzymes is inconclusive without accompanying evidence that the recombinant E2 enzymes used were, in fact, active.

We agree that a control is required here and have now performed E2 charging assays with each E2 used demonstrating that they can all be charged with ubiquitin and are therefore active. These new data are presented as new Figure E4

6. The identification of the TRIM5α lysines (i.e., K45 & K50) targeted for

autoubiquitination provides an obvious opportunity to test the relevance of those sites for TRIM5α ubiquitination in cells and for their role in viral restriction. Those experiments are well within the scope of this study and should be done.

We have made 2 pairs of TRIM5α mutants, the first mutated at the major Ub targets at positions 45 and 50, plus the adjacent lysine at position 44. We also made a second pair of mutants mutated at all of the lysine residues found to be targeted by ubiquitin by MS. These mutants have been used in in vitro ubiquitination assays and in cellular restriction studies presented as new Figures 7 and E8 and described on pages 13 and 14.

Some minor issues:

7. In Fig. 4E, "RLU" should be defined.

RLU is now defined in the figure legend

8. There are typos in many of the concentration units in Fig. 5

These have now been corrected

9. The magnified panel D in Fig. 3 is unnecessary.

We have now removed this panel

10. (p. 8, line 35) Contrary to the claim, the ESI mass spectrum does not show definitively that 'demethionylation' and N-acetylation were 100%.

We appreciate this point and have changed the text to "Electrospray mass spectrometric analysis of the intact purified recombinant TRIM5α_{rh} protein indicated that demethionylation and *α*N-acetylation were complete because species representing methionylated or demethionylated and non-acetylated forms of TRIM5α_{rh} were not detected (Fig. E3). Thus, as far as we could tell, our recombinant TRIM5α_{rh} protein had the same N-terminal sequence and modification pattern as TRIM5α proteins expressed in human 293T cells."

11. (p. 9, lines 1-5) The Pickart & Raasi paper does not describe purification of most of the E2 enzymes used by Fletcher et al. The sentence here is ambiguous.

We agree. We have reworded to clarify that the Raasi paper refers to ubiquitin purification and E1, E2 purifications are described in the method section. New text "Ub (Pickart & Raasi, 2005), E1 (UBA1) and E2 enzymes were all expressed in *E. coli* and purified to homogeneity for use in the *in vitro* ubiquitination assays, see Methods section

Referee #2:

In this manuscript Fletcher and colleagues performed a targeted siRNA screen and show that the E2 enzymes Ube2W and Ube2N/Ube2V2 are required to inhibit N-MLV reverse transcription by human TRIM5α. The authors further propose a two-step ubiquitination mechanism in which Ube2W-dependent TRIM5α mono-ubiquitination is required for subsequent K63-linked TRIM5α polyubiquitination by the E2 Ube2N/Ube2V2 and TRIM5α-mediated suppression of viral DNA synthesis.
These findings have a significant impact on the field since the molecular mechanism of TRIM5α restriction, and in particular the involvement of TRIM5α ubiquitination in retrovirus restriction still remain poorly defined and somewhat controversial. The

© European Molecular Biology Organization

experiments are convincingly performed and clearly presented. However the authors should address the following points to confirm some of their findings.

Specific comments:

1) Does the inhibition of DNA synthesis shown in Fig. 1C correlate with virus induced degradation of TRIM5α?

No, we do not see virus induced degradation of TRIM5 although this has been previously described by the Aiken lab. We didn't focus on this question but imagine that differences in experimental set up, particularly in levels of TRIM5 expression could underlie this result. We feel that addressing this question experimentally in a conclusive way is unlikely to shed further light on the mechanism of TRIM5 ubiquitination and is beyond the scope of the current study.

2) In Fig 2 the authors convincingly show that ectopically expressed TRIM5α is ubiquitinated and rapidly turned over by the proteasome. Can the authors provide any endogenous data to support this finding? Is the HMW TRIM5α which accumulates upon MG132 treatment (Fig. 2A) also detected by a K63UB specific antibody?

Despite testing TRIM5α antibodies we have been unable to perform this experiment with endogenous TRIM5α due to poor specificity/reactivity of these reagents. We have shown that we can purify ubiquitinated TRIM5α in the absence of MG132 and that this TRIM5 can be detected by the K63R Ub Ab (Fig 4C). Although this does not test whether K63 ubiquitinated TRIM5 accumulates on proteasome inhibition it clearly shows that K63 ubiquitinated TRIM5 can be detected in cells. Furthermore, the fact that HMW TRIM5 species are lost on Ube2N depletion (Fig. 3B and 4B) suggests they are K63-enriched.

3) By mass spectrometry analysis the authors identified Lys45 and Lys50 as the residues on TRIM5α that are ubiquitinated on a Ube2W-dependent fashion. The authors should address ubiquitination status and antiretroviral activity of a TRIM5α protein mutated at these sites.

Reviewer #1 raised the same point, and our answer is the same.

Referee #3:

This manuscript studies TRIM5alpha mediated antiviral activity. Using an siRNA screen the authors identify the E2 enzymes UBE2W and UBE2N/UBE2V2 (ubc13/mms2) as important for the ability of TRIM5a to abrogate reverse transcription, but not for its effect on infectivity.

They then show that these E2s are required to modify TRIM5alpha itself with UBE2W priming and Ubc13/mms2 extending the k63 chain. That is interesting as the UBE2W enzyme is known to modify N-terminal residues, whereas TRIM5alpha is acetylated at its N-terminus, preventing this modification. They then nicely show biochemically that UBE2W can place two ubiquitins at residues 45 and 50 if it is acetylated, and a third, when it is not.

The experiments are well-executed and the manuscript is well-written. To identify correct E2/E3 combinations is still quite a feat, and the data in this manuscript are convincing that the link between TRIM5a and these E2s is real. Moreover, the ability of Ube2w to do more

than target N-termini is very intriguing and will be important for understanding how this E2 functions.

What is missing is a functional link between the auto-ubiquitination of the ligase and its role in antiviral activity. At the very least the two acceptor lysines in Trim5alpha should be mutated, to see if it does play a role.

Reviewers #1 and raised the same point, and our answer is the same.

If that role is present, it would of course be very interesting to see what the K63 chains would bind to, but this may beyond the scope of the paper. However, if there is no functional link, one wonders what the target might be.

Unfortunately we have not been able to establish how the K63 chains lead to restriction of DNA synthesis. We argue that this significant extension of the discoveries described is beyond the scope of the current manuscript.

Minor points:

Page 8, line 22: unpublished observations? This does not seem appropriate, please just include the data here?

These data are now included as a new Fig E3

It is probably helpful to mention that UBE2N/UBE2V2 are UBC13/MMS2.

*We have stated in the text "*The three strongest hits were Ube2W, Ube2N (Ubc13) and Ube2V2 (Mms2)*"*

2nd Editorial Decision 12 May 2015

Thank you for submitting your revised manuscript to The EMBO Journal. Your study has now been re-reviewed by referees #1 and 3.

The revised version received a bit of a mixed response. While referee #3 is supportive of the revision version pending some text changes, referee #1 is not convinced that the manuscript sufficiently show that TRIM5α autoubiquitination is important for viral restriction.

I see the point that referee #1 makes, but I also see the value of the system and the insights gained into TRIM5α's function and antiviral activity. I am therefore pleased to accept the paper for publication here. Before formal acceptance here, I would like to ask you to incorporate referee #3's suggestions and also to respond to the concerns raised by referee#1 (be it in the point-by-point response or the discussion).

You can use the link below to upload the revised version. If you have any further questions please don't hesitate to contact me.

REFEREE REPORTS

Referee #1:

© European Molecular Biology Organization

Fletcher et al. have done a good job addressing most of the technical problems identified by the reviewers, but the revised manuscript nonetheless fails to answer a key issue raised in the original reviews: does TRIM5α's autoubiquitination have functional significance for viral
restriction? In my view, the significance of the work depends greatly on this point; unfortunately, despite the new experiments done to address this (i.e., mutations in TRIM5alpha to limit its autoubiquitination), the results are inconclusive. In part, this is because TRIM5alpha was found to be promiscuous with respect to the sites of its autoubiquitination. However, even when the authors effectively reduced autoubiquitination via multiple K-to-R substitutions and observed a coincident loss of restriction, a solid interpretation was not possible because of the possibility that the mutations interfered with TRIM5alpha binding to the viral capsid protein (p. 13-14). Thus, whether TRIM5alpha autoubiquitination is important for viral restriction remains unclear. Because this question is central to their paper, I am very surprised that the authors did not examine capsid protein binding by the mutant versions of TRIM5alpha. In citing Sebastian et al. 2009 (p. 14), Fletcher et al. seem to suggest that the K-to-R mutations they made in the PRYSPRY loop5 sequence reduced restriction because of defects in capsid binding. In fact, the Sebastian et al. study had used substitutions by alanine, which are most likely much more severe than the K-to-R changes made in the current work. In any case, the effects on capsid binding should have been examined directly by Fletcher et al. to help eliminate this ambiguity in interpreting the result.

Referee #3:

This is still a very nice dissection of a complex chain formation system that is important for defense against viruses. The screen is really nice, as it is so black and white and data are nicely done. The ability to counterscreen with the two different viruses is very useful and the clarity of results is great.
Beyond its specific importance for TRIM5a function and antiviral infection, the manuscript is certainly interesting to the ubiquitin field for its careful analysis of the E2W function, showing that this is capable of aminoterminal conjugation on TRIM5a, but does not in practice do so due to its N-acetylation.

The problem indicated by reviewer 1, that the proteasome inhibitor mg132 does not cause depletion of the unmodified TRIM5a, despite the increase in modified versions must surely indicate that total levels go up as a result of the mg132 treatment. This is most likely a different point than the chain formation studied here, but could be discussed in more detail.

Of course It is a pity that the site-specificity is not enough to allow validation by mutation, but this is not uncommon for ubiquitin pathways. The explanation why use of alternative sites is is more evident in cells than in vitro could surely also be due to presence of additional E3 ligases that promote the reactions. It could be good to add this option to the discussion.

2nd Revision - authors' response — 18 May 2015

Point by point rebuttal for "TRIM5α requires Ube2W to anchor Lys63-linked ubiquitin chains and restrict reverse transcription" EMBOJ-2014-90361

Referee #1:

Fletcher et al. have done a good job addressing most of the technical problems identified

by the reviewers, but the revised manuscript nonetheless fails to answer a key issue raised in the original reviews: does TRIM5α's autoubiquitination have functional significance for viral
restriction? In my view, the significance of the work depends greatly on this point; unfortunately, despite the new experiments done to address this (i.e., mutations in TRIM5alpha to limit its autoubiquitination), the results are inconclusive. In part, this is because TRIM5alpha was found to be promiscuous with respect to the sites of its autoubiquitination. However, even when the authors effectively reduced autoubiquitination via multiple K-to-R substitutions and observed a coincident loss of restriction, a solid interpretation was not possible because of the possibility that the mutations interfered with TRIM5alpha binding to the viral capsid protein (p. 13-14). Thus, whether TRIM5alpha autoubiquitination is important for viral restriction remains unclear. Because this question is central to their paper, I am very surprised that the authors did not examine capsid protein binding by the mutant versions of TRIM5alpha. In citing Sebastian et al. 2009 (p. 14), Fletcher et al. seem
to suggest that the K-to-R mutations they made in the PRYSPRY loop5 sequence reduced restriction because of defects in capsid binding. In fact, the Sebastian et al. study had used substitutions by alanine, which are most likely much more severe than the K-to-R changes made in the current work. In any case, the effects on capsid binding should have been examined directly by Fletcher et al. to help eliminate this ambiguity in interpreting the result.

We appreciate this reviewer's enthusiasm for our addressing the technical problems identified. However, we do not think that examining whether the TRIM5 lysine mutations influence TRIM5 binding to capsid in *in vitro* binding assays will clarify mechanistic details of TRIM5 ubiquitination. This is because we have shown that the 7KR mutations prevent restriction of viral infection. Knowing whether the 7KR mutant still bound the capsid in *in vitro* assays would not help us understand whether the mutations had disturbed the process of restriction of infection in a more subtle way. Our experiments have established that preventing TRIM5 ubiquitination by E2 depletion or K63R Ub expression, (Fig 1-2) rescues viral DNA synthesis but not infection. Thus we know that ubiquitination is not required for restriction of infection and we can cleanly separate the effects of TRIM5 binding (restriction of infection) and ubiquitination (restriction of DNA synthesis). We can therefore only reliably interpret the results using the 7KR mutant if it gives this same clean result, impacting restriction of DNA synthesis, but not infection. Unfortunately, mutations including those at positions 367 and 368 in the PRYSPRY impact restriction of DNA synthesis **and** infection (Fig E8 A, B). Therefore we do not know whether we have lost restriction of DNA synthesis because we have lost restriction of infection altogether or because we have lost the critical ubiquitination of TRIM5. Importantly, the TRIM5 7KR mutant is still ubiquitinated in cells (Fig E8 C, D) suggesting that, in fact, we have not lost restriction through loss of ubiquitination. Rather, we have lost the ability to restrict infection entirely through mutating the TRIM5-capsid interface. We therefore argue that the loss of restriction by TRIM5 7KR prevents resolution of this question within the scope of this study.

Referee #3:

This is still a very nice dissection of a complex chain formation system that is important for defense against viruses. The screen is really nice, as it is so black and white and data are nicely done. The ability to counterscreen with the two different viruses is very useful and the clarity of results is great.
Beyond its specific importance for TRIM5a function and antiviral infection, the manuscript is certainly interesting to the ubiquitin field for its careful analysis of the E2W function, showing that this is capable of aminoterminal conjugation on TRIM5a, but does not in practice do so due to its N-acetylation.

The problem indicated by reviewer 1, that the proteasome inhibitor mg132 does not cause depletion of the unmodified TRIM5a, despite the increase in modified versions must surely indicate that total levels go up as a result of the mg132 treatment. This is most likely a different point than the chain formation studied here, but could be discussed in more detail.

It is indeed our interpretation that total TRIM5 levels rise on MG132 mediated proteasome inhibition and that this is due to inhibition of degradation of the HMW modified TRIM5 species. We have clarified the text adding the italicized sentence.

Measurement of the HA band densities corresponding to the unmodified and HMW forms of HA-TRIM5α_{hu} (of Fig 2A) demonstrated that it is not the unmodified form that accumulated in these experiments (Fig. 2A, bottom), rather it is the HMW species that accumulate, consistent with post-translational modification, such as ubiquitination. "*We assume their accumulation reflects a lack of degradation owing to proteasome inhibition."*

Of course it is a pity that the site-specificity is not enough to allow validation by mutation, but this is not uncommon for ubiquitin pathways. The explanation why use of alternative sites is more evident in cells than in vitro could surely also be due to presence of additional E3 ligases that promote the reactions. It could be good to add this option to the discussion.

We agree and have added the following to the discussion:

"Finally, it is possible that other E3 ligases functionally ubiquitinate TRIM5α via the E2 enzymes identified here. However, our observation that TRIM5α is able to autoubiquitinate in vitro using these enzymes makes TRIM5α itself the most likely E3 candidate for TRIM5α-dependent restriction.."

© European Molecular Biology Organization
